# MiR-9 promotes tumorigenesis and angiogenesis and is activated by MYC and OCT4 in human glioma

**DOI:** 10.1186/s13046-019-1078-2

**Published:** 2019-02-22

**Authors:** Xu Chen, Fan Yang, Tianze Zhang, Wei Wang, Wenjin Xi, Yufang Li, Dan Zhang, Yi Huo, Jianning Zhang, Angang Yang, Tao Wang

**Affiliations:** 1 State Key Laboratory of Cancer Biology, Department of Immunology, Fourth Military Medical University, #169 Changle West Road, Xi’an, Shaanxi 710032 People’s Republic of China; 2 Department of Neurosurgery, General Navy Hospital of PLA, Beijing, 100048 People’s Republic of China; 3 Nuclear Medicine Diagnostic Center, Shaanxi Provincial People’s Hospital, Xi’an, Shaanxi 710032 People’s Republic of China; 4 First Student Brigade, Fourth Military Medical University, Xi’an, Shaanxi 710032 People’s Republic of China; 5 Department of Medical Genetics and Developmental Biology, Fourth Military Medical University, #169 Changle West Road, Xi’an, Shaanxi 710032 People’s Republic of China

**Keywords:** miR-9, Glioma, Tumorigenesis, Angiogenesis, MYC, OCT4

## Abstract

**Background:**

Glioma, characterized by its undesirable prognosis and poor survival rate, is a serious threat to human health and lives. MicroRNA-9 (miR-9) is implicated in the regulation of multiple tumors, while the mechanisms underlying its aberrant expression and functional alterations in human glioma are still controversial.

**Methods:**

Expressions of miR-9 were measured in GEO database, patient specimens and glioma cell lines. Gain- and loss-of-function assays were applied to identify the effects of miR-9 on glioma cells and HUVECs in vitro and in vivo. Potential targets of miR-9 were predicted by bioinformatics and further verified via in vitro experiments. Transcriptional regulation of miR-9 by MYC and OCT4 was determined in glioma cells.

**Results:**

MiR-9 was frequently up-regulated in glioma specimens and cells, and could significantly enhance proliferation, migration and invasion of glioma cells. In addition, miR-9 could be secreted from glioma cells via exosomes and was then absorbed by vascular endothelial cells, leading to an increase in angiogenesis. COL18A1, THBS2, PTCH1 and PHD3 were verified as the direct targets of miR-9, which could elucidate the miR-9-induced malignant phenotypes in glioma cells. MYC and OCT4 were able to bind to the promoter region of miR-9 to trigger its transcription.

**Conclusions:**

Our results highlight that miR-9 is pivotal for glioma pathogenesis and can be treated as a potential therapeutic target for glioma.

**Electronic supplementary material:**

The online version of this article (10.1186/s13046-019-1078-2) contains supplementary material, which is available to authorized users.

## Background

Human glioma, encompassing a heterogeneous cluster of subtypes, accounts for 50–60% of the primary central nervous system (CNS) tumors in adults [1]. High-grade glioma, especially the most lethal pathological subtype, glioblastoma, causes a serious decline of 2-year survival rate to only 26% [2]. Despite some certain efficacy of the cytoreductive surgery in combination with the intense chemoradiotherapy, glioma still harbors an undesirable prognosis [3] and a poor median survival phase of 14.6 months [4]. Tumorigenesis and angiogenesis are two pivotal aspects for glioma development. Glioma tumorigenesis significantly elevates the intra-glioma microvessel density, and angiogenesis is critical for glioma growth, migration and invasion [5]. Though various genetic and epigenetic regulations synergistically contribute to the tumorigenesis and angiogenesis of glioma, the key element triggering or inhibiting these processes is still largely unknown [6]. Therefore, characterization of sensitive targets for tumorigenesis and/or angiogenesis is urgently needed to illustrate the molecular events involved in glioma initiation, development and progression.

MicroRNAs (miRNAs) are an enormous group of small non-coding RNAs that suppress nearly 1/3 of the human genes at post-transcriptional or translational level by binding to the complementary sequences in 3′-untranslated region (3’-UTR) of target mRNAs [7, 8]. MiRNAs have been reported to mediate abundant biological processes, including tumorigenesis, while the precise effects of miRNAs on human glioma and reasons for their dysregulation remain largely unknown. Recently, several miRNAs were identified as onco-miRs or tumor suppressors in glioma [9–12]. These miRNAs, accompanied by their target genes, comprise an intricate network that modulates the pathology and physiology of glioma.

MiR-9 was originally reported to be positively correlated with neurogenesis [13]. Studies have shown aberrant expression of miR-9 in different tumors, whereas the role of miR-9 in tumors appears to be controversial, as a pro-metastasis onco-miR in the breast cancer [14, 15] but a tumor suppressor in the melanoma [16]. Meanwhile, miR-9 can combine neurogenesis with angiogenesis [17] and exert a dual-effect upon regulation of lymphatic inflammatory and lymphangiogenic pathways [18], indicating that miR-9 is potentially associated with the generation of new vasculature. However, the functions of miR-9 in glioma angiogenesis and the molecular mechanisms through which miR-9 influences the malignant phenotypes of glioma cells, have not been fully understood until now.

In this study, we confirm that miR-9 is frequently up-regulated in glioma patients and cells. Glioma and HUVEC cell proliferation, migration, invasion and new vessel formation are significantly enhanced by miR-9 both in vitro and in vivo. In addition, miR-9 inhibits the expression of COL18A1, THBS2, PTCH1 and PHD3 and promotes transduction of HIF-1α/VEGF signaling pathway. Furthermore, MYC/OCT4 directly binds to the promoter region of miR-9-2 and launches its transcription. Taken together, our findings provide valuable clues regarding the mechanism of glioma pathogenesis and offer a promising target for the future development of effective therapies.

## Methods

### Glioma patient specimens

Glioma patient specimens (*n* = 18) and normal tissues (*n* = 3) were acquired from Neurosurgery Department of Xijing Hospital affiliated to the Fourth Military Medical University (FMMU). Patient samples were pathologically evaluated and immediately frozen in liquid nitrogen. Informed consent was obtained from all the individuals, and experimental protocols were approved by the Medical Ethics Committee of Xijing Hospital. Detailed information on the 18 tissues of glioma patients is listed in Additional file 1: Table S1.

### Cell cultivation

Human glioma A172, U251, U87, BT325 and SHG44 cell lines were purchased from the Neurosurgery Department of Xijing Hospital and cultured in high glucose Dulbecco’s modified Eagle’s medium (DMEM, Gibco, Los Angeles, USA). Normal glia HEB cells were obtained from the Institute of Biotechnology Research (Beijing, China) and maintained in low glucose DMEM (Gibco). HUVECs and HEK293T cells were cultured in DMEM (Gibco). Murine GL261 cells were maintained in Roswell Park Memorial Institute medium-1640 (RPMI-1640, Gibco). All the above cells were incubated at 37 **°**C with 5% CO_2_ and cultivated in the medium supplemented with 10% fetal bovine serum (FBS, Invitrogen, Carlsbad, USA), 100 U/mL penicillin and 0.1 mg/mL streptomycin.

### Transient transfection and lentivirus infection

Transient transfection of the indicated oligonucleotides was performed by using Lipofectamine 2000 Transfection Reagent (Invitrogen) according to manufacturer’s instructions. Lentiviruses were produced, purified and concentrated in the Genecopies (Guangzhou, China). GL261 cells were stably infected with the miR-9-overexpressing (LV-miR-9) or negative control (LV-NC) lentiviral particles, respectively. Polybrene (8 μg/mL) (Millipore, Billerica, USA) was also added. Information on the sequences of specific oligonucleotides is listed in Additional file 1: Table S2.

### Gene expression analysis

Total RNA, including mRNA and miRNA, was extracted from the glioma cells by TRIzol reagent (Invitrogen). Each RNA sample was quantified and subjected to the reverse transcription. The mRNA and miRNA reverse transcription reactions were performed with the PrimeScript™ RT Master Mix and SYBR® PrimeScript™ miRNA RT-PCR Kit (TaKaRa, Shiga, Japan), respectively. Expression levels of the reverse transcribed templates were determined by the quantitative real-time polymerase chain reaction (qRT-PCR) with FastStart Essential DNA Green Master (Roche, Indianapolis, USA) according to the manufacturer’s protocols. Beta-actin and U6 were regarded as the mRNA and miRNA normalization controls, respectively. Each experiment was performed in triplicate, and fold changes were calculated by the relative quantification method (2 ^-∆∆CT^). The qRT-PCR primers are listed in Additional file 1: Table S3.

### Protein expression analysis

Cells in the 6-well plates were lysed in 100–150 μL of the RIPA lysis buffer supplemented with 1% phenylmethylsulfonyl fluoride (PMSF, Beyotime, Shanghai, China). Protein samples were quantified via the bicinchoninic acid (BCA) method (Thermo, Massachusetts, USA) and separated in an 8–10% SDS-PAGE gel (Invitrogen) at a constant voltage. Next, proteins were transferred onto a polyvinylidene fluoride (PVDF) membrane (Millipore) at a constant current. After the PVDF membranes were blocked for 1 h in the 5% bovine serum albumin (BSA), they were incubated with indicated primary antibodies at 4 °C overnight. Then, membranes were washed several times with TBST (Tris-buffered saline with 0.05% Tween-20), followed by incubation with secondary antibodies at room temperature for 1 h. Finally, the membranes were analyzed on FluorChem FC2 system (Alpha Innotech, San Leandro, USA). Detailed information on the antibodies is listed in Additional file 1: Table S4.

### Cell co-culture system

For cell co-culture system, 5 × 10^5^ HUVEC cells were re-suspended in 500 μL serum medium and then seeded into the upper transwell chamber with a non-coated membrane (24-well insert; pore size, 0.4 μm; Millipore). Meanwhile, 5 × 10^5^ glioma cells with or without miR-9 transfection were incubated in the lower chamber with 500 μL serum medium. After co-cultivation at 37 **°**C with 5% CO_2_ for indicated time, the HUVEC cells in the upper chamber were harvested for further detection.

### Enzyme-linked immuno sorbent assay (ELISA)

5 × 10^5^ HUVECs were co-cultured with A172 miR-9 mimic/NC cells and U251 miR-9 inhibitor/NC cells in 24-well flat-bottom for 72 h, respectively. Endostatin in conditional medium as well as cell lysates and VEGF in cell lysates were determined by an ELISA kit (Dakewe Biotech, Shenzhen, China) according to the manufacturer’s instructions. Absorbance at 450 nm was measured using the multi-well plate reader (Bio-Rad, Hercules, USA).

### Cell adhesive force assay

HUVEC cells (4 × 10^3^ per well, four groups according to time spots) were seeded in the 96-well plate coated with 50 μL of Matrigel gel and maintained at 37 °C with 5% CO_2_ for 0.5, 1, 2 and 4 h, respectively. At each time spot, supernatant of the corresponding group was entirely discarded and the cells within were washed with phosphate-buffered saline (PBS) for three times. Then, cells were fixed with 4% paraformaldehyde, stained by 0.5% crystal violet and blended with 100 μL of 11% acetic alcohol for 10 min. Absorbance at 570 nm was measured using the multi-well plate reader (Bio-Rad). Each experimental group was performed in sextuplicate.

### Bioinformatics study

Potential targets of miR-9 were predicted by four computer-aided algorithms: Targetscan (http://www.targetscan.org/), PicTar (http://pictar.mdc-berlin.de/), microRNA (http://www.microrna.org/microrna/getMirnaForm.do) and miRbase (http://www.mirbase.org/). Transcription factors (MYC and OCT4) and their binding sites were predicted by the UCSC (http://genome.ucsc.edu/).

### Dual-luciferase reporter assay

The wild-type (WT) or mutant (MuT) 3′-UTRs of COL18A1, THBS2, PTCH1 and PHD3 were amplified by PCR and cloned into the multiple cloning site (MCS) of PGL3-MCS2 miRNA firefly expression reporter vector. 1 × 10^4^ HEK293T cells were seeded in the 48-well plates and co-transfected with the wild-type or mutant luciferase reporter (100 ng), miR-9 mimics (20 nM) and NCs as indicated. Renilla luciferase vector (20 ng) was used for normalization. After 48 h, the relative luciferase activity was detected by a dual-luciferase reporter system (Promega, Madison, USA). Final results are shown as the ratio of firefly to Renilla fluorescence intensity. The wild-type and mutant PCR primers are listed in Additional file 1: Table S5.

### Cell proliferation analysis

First, 2 × 10^3^ glioma cells were seeded into the 96-well plates with 200 μL of medium (DMEM) supplemented with 10% FBS per well. Twenty microliters of the 3-(4, 5-dimethylthiazol-2-yl)-2, 5-diphenyltetrazolium bromide (MTT, Sigma-Aldrich, Santa Clara, USA) was added to each well, and cells were incubated for 4 h. Then, 150 μL of the dimethyl sulfoxide (DMSO) was used to dissolve the insoluble products after removing the supernatant. Absorbance (570 nm) was measured at indicated time (0, 24, 48, 72 and 96 h) using a multi-well plate reader (Bio-Rad). Each experimental group was performed in sextuplicate.

### Cell cycle and apoptosis analysis

Flow cytometry (FCM) was applied to explore the cell cycle and apoptosis. For cell cycle analysis, cells were washed thrice with PBS, fixed with 70% ethanol and stored at 4 °C overnight. Then, fixed cells were washed twice with PBS and stained by PE-conjugated propidium iodide (PI) for 2 h at 4 °C in the darkness. For apoptosis analysis, pre-treated glioma cells were harvested and washed thrice with PBS. Then, cells were incubated with 100 μL of RNase (100 mg/L) for 30 min at 37 °C and then stained with PI and annexin V-FITC at 4 °C for 30 min. The cell cycle and apoptosis were monitored at 488 nm. Each sample containing 1 × 10^6^ cells was performed in triplicate.

### Cell migration and invasion analysis

Briefly, for transwell migration assay, 1 × 10^5^ cells were plated into the upper chamber with a non-coated membrane (24-well insert; pore size, 8 μm; Millipore). For transwell invasion assay, 3 × 10^5^ cells were plated into the upper chamber with a Matrigel-coated membrane. In both assays, experimental cells were re-suspended in serum-free medium, and 500 μL of medium supplemented with 10% FBS was added into the lower chamber. Cells were incubated for 24–36 h at 37 °C. Cells that did not migrate or invade through the pores were removed using a cotton swab. Invading or migrating cells were then fixed with 4% paraformaldehyde, stained with 0.1% crystal violet and counted under a light microscopy. For wound-healing assay, 5 × 10^5^ cells were plated and cultured to 90% confluence in a 6-well plate. A 100 μL pipette tip was used to vertically scratch the monolayer of cells, thereby creating a wound, and photos were taken at indicated time (0, 24 and 48 h) under a microscope. Each experiment was performed at least in triplicate.

### Capillary-like tube formation assay

Firstly, 1.5 × 10^4^ HUVECs were cultivated in conditional medium for 6 h at 37 °C in a 48-well plate coated with 200 μL Matrigel. Formation of capillary-like structures was captured under a light microscopy. Branch points of the newly formed tubes were scanned and quantified in five random low-power fields. Each assay was performed in triplicate.

### Purification and identification of exosome

80% confluent A172/U251 cells were washed with PBS three times and cultured in the Exo-Clear Cell Growth Medium (SBI, Palo Alto, USA) for 72 h. Conditional medium of A172/U251 cells was then filtered using a 0.45 μm Steritop™ (Millipore). For exosome purification, conditional medium was transferred to a sterile ultra-clear tube and centrifuged at 120,000 g for 75 min at 4 °C. The pelleted precipitates were re-suspended in 1 mL PBS and ultra-centrifuged at 120,000 g for 75 min. Exosomes were re-suspended in 100 μL PBS and stored at − 80 °C for further experiments. Total RNAs in the exosomes were extracted using the Qiagen miRNeasy Serum/Plasma Kit (Qiagen, Valencia, USA) according to manufacturer’s instruction. For identification of exosomes, purified exosomes were negatively stained with 2% phosphotungstate for 3 min. Next, exosomes were loaded on a copper grid and visualized by a transmission electron microscope (JEOL, Tokyo, Japan). The photos were obtained with a digital camera (Olympus, Tokyo, Japan).

### Animal study

These in vivo experiments were approved by the Institutional Animal Care and Use Committee of FMMU, and were strictly implemented according to institutional guidelines.

For intracranial xenograft, female 6–8 weeks old C57BL/6 mice were randomly divided into five per group and stabilized by a stereotaxic apparatus (KOPF940). Then, GL261 LV-miR-9/NC cells were injected into the right frontal lobe of brain with 5 × 10^6^ cells in 20 μL of saline. After 2 weeks, the mice were sacrificed and their brains were taken out to perform hematoxylin and eosin (H&E) and immunohistochemistry (IHC). Another intracranial xenograft assay was used to examine the tumor size and overall survival of the experimental mice. Tumor size was calculated according to the following formula: V = (W^2^ x L) / 2, W < L [19]. Weights of the mice were measured every 3 days and overall survival days of the mice were counted.

For subcutaneous xenograft, female 6–8 weeks old athymic BALB/c mice were injected with GL261 LV-miR-9/NC cells into the dorsal flanks of each athymic mouse (5 × 10^6^ cells/injection, five mice per group). Tumor size was detected every 5 days and was calculated as follows: V = (W^2^ x L) / 2, W < L. Finally, mice were sacrificed, and the neoplasms were fixed in 4% paraformaldehyde. Tissue sections were stained by H&E and IHC.

### Chromatin immunoprecipitation (ChIP)

Chromatin immunoprecipitation (ChIP) assay was depended on manufacturer’s instructions from the Chromatin Immunoprecipitation Assay Kit (Millipore). Briefly, DNAs were firstly cross-linked and cells were re-suspended in the RIPA lysis buffer supplemented with protease inhibitor cocktail (Roche). Then, DNAs were sheared by sonication into 100–200 bp fragments. Appropriate primary antibodies and magnetic beads were incubated with sonicated DNA fragments at 4 °C overnight. Beads were washed multiple times with RIPA lysis buffer, DNA cross-links were chelated, and target DNA fragments were precipitated by 70% ethanol. DNA samples were detected by qRT-PCR using five primers that were within the − 2500 bp to + 500 bp region of the miR-9-2 promoter. ChIP primers are listed in Additional file 1: Table S6.

### Statistical analysis

Experimental data were analyzed by SPSS 18.0, and the results are presented as the mean ± standard deviation (s.d.). Statistical significance between two individuals or groups was evaluated by Student’s t-test. Analysis with more than two groups was determined by one-way ANOVA test. Overall survival of the experimental group was analyzed by the Kaplan-Meier survival curve. The results were considered significant at *P < 0.05, **P < 0.01 and ***P < 0.001.

## Results

### MiR-9 is aberrantly expressed and positively correlated with cell proliferation, invasion and migration in human glioma

**Fig. 1:**
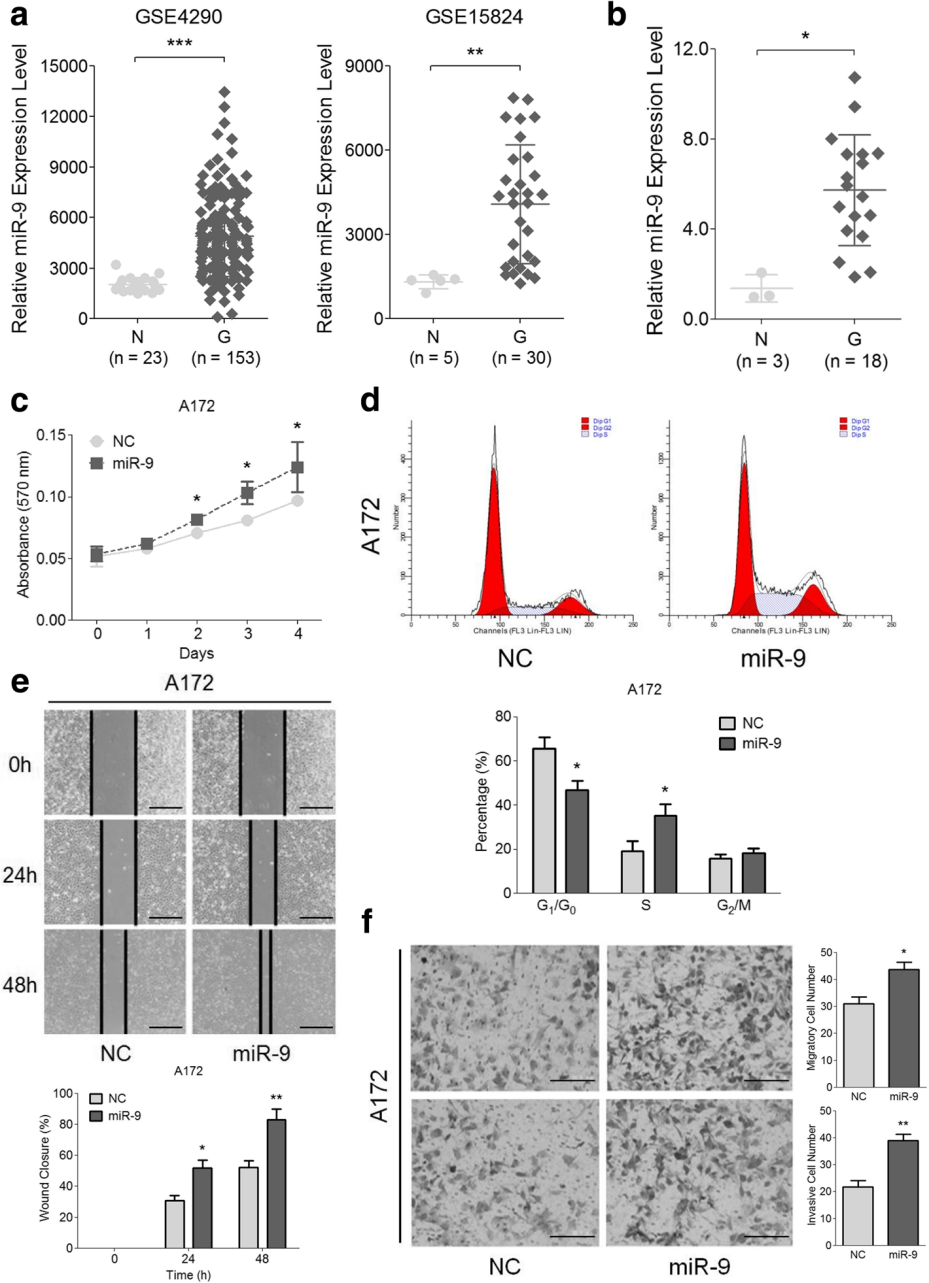
MiR-9 is frequently up-regulated in glioma tissues and facilitates glioma cell proliferation, migration and invasion. **a** MiR-9 expression levels were analyzed in the data from GSE4290 (N, *n* = 23; G, *n* = 153; *left*) and GSE15824 (N, *n* = 5; G, *n* = 30; *right*), respectively. N, normal tissues; G, glioma tissues. Data are represented as the mean ± s.d. (**P < 0.01 and ***P < 0.001). **b** MiR-9 expressions in the 3 normal (N) and 18 glioma (G) tissues were detected by qRT-PCR. Data are shown as the mean ± s.d. (*P < 0.05). **c** Proliferation of the A172 miR-9 mimic/NC cells was measured by MTT assay. Error bars represent the s.d. (*P < 0.05; *n* = 6 independent experiments). **d** Flow cytometry analysis was used to evaluate cell cycle of A172 miR-9 mimic/NC cells. Data are shown as the mean ± s.d. (*P < 0.05; *n* = 3 independent experiments). **e** Wound-healing assay was used to determine the migration of A172 miR-9 mimic/NC cells (*upper*). Photos were taken at 0, 24 and 48 h, respectively. Histogram was used for the statistical analysis of wound-healing assay for A172 miR-9 mimic/NC cells (*lower*). *Scale bars* represent 200 μm. Data are represented as the mean ± s.d. (*P < 0.05 and **P < 0.01; *n* = 3 independent experiments). **f** Migration (*upper*) and invasion (*lower*) of the A172 miR-9 mimic/NC cells were measured by the non-coated transwell and Matrigel-coated transwell assays, respectively. *Scale bars* represent 100 μm. Data are shown as the mean ± s.d. (*P < 0.05 and **P < 0.01; *n* = 3 independent experiments)

To better understand the potential connection between miR-9 and human glioma, we explored the expression levels of miR-9 in Gene Expression Omnibus (GEO) database, clinical specimens and glioma cell lines. As shown in Fig. 1a, both of the GSE4290 and GSE15824 experiments showed that the expression of miR-9 in glioma (G) was significantly higher than that in control tissues (N). Consistent with this, the expression of miR-9 in tissues from 18 glioma patients (G) also increased compared with normal control tissues from 3 healthy volunteers (N) (Fig. 1b and Additional file 2: Figure S1a). However, no significant differences in miR-9 expression were detected in the tissues which were classified in accordance with the WHO grade in GSE4290 and the specimens we collected (Additional file 2: Figure S1b). We also determined the endogenous miR-9 levels in five glioma cell lines (A172, SHG44, U87, BT325 and U251) and a normal glia cell line (HEB). Consistent with Fig. 1a and b, the expression of miR-9 was increased in all five glioma cell lines compared with HEB cells (Additional file 2: Figure S1c). Taken together, these data provided clues to build a potential positive correlation between miR-9 and human glioma.

Next, we explored the potential role of miR-9 involved in glioma tumorigenesis. For this purpose, we performed gain- and loss-of-function assays to examine the effects of miR-9 in A172 and U251 cells on different biological behaviors (Additional file 2: Figure S1d). Cell proliferation was analyzed by MTT assays and flow cytometry in vitro. MiR-9 overexpression was correlated with a notable increase in cell growth (Fig. 1c) and an accelerated progression in cell cycle (Fig. 1d); conversely, knocking down miR-9 decreased the proportion of proliferating cells and inhibited cell cycle (Additional file 2: Figure S2a and S2b). However, there were no significant differences in cell apoptosis between miR-9-treated or NC-treated cells (Additional file 2: Figure S2c). In wound-healing and transwell assays, miR-9 overexpression promoted A172 cells to heal the scratched wound (Fig. 1e), but miR-9 knockdown prevented U251 cells from generating a complete monolayer (Additional file 2: Figure S2d). In addition, up-regulating miR-9 prompted more A172 cells to migrate to the lower chamber and to invade through the Matrigel (Fig. 1f), whereas down-regulating miR-9 significantly inhibited the migration and invasion of U251 cells (Additional file 2: Figure S2e). Collectively, these data suggested that miR-9 was able to promote cell proliferation and cell cycle progression and could significantly enhance invasion and migration in glioma cells in vitro.

### MiR-9 strengthens the angiogenesis functions of HUVECs

**Fig. 2:**
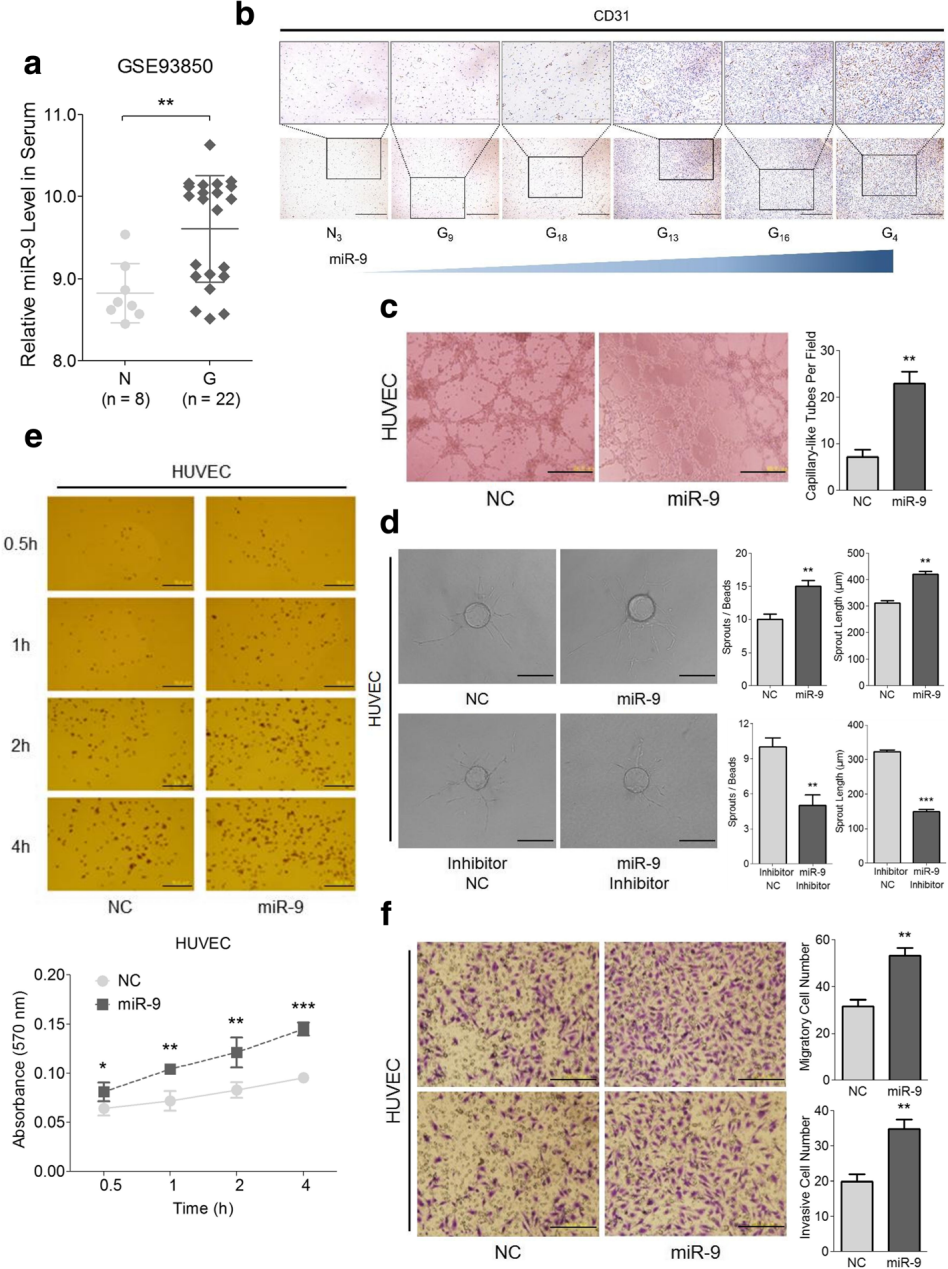
MiR-9 promotes angiogenesis, adhesion, migration and invasion of HUVECs. **a** MiR-9 expression levels in the serum of normal individuals (N, *n* = 8) and glioma patients (G, *n* = 22) were analyzed by using the data from GSE93850. Data are shown as the mean ± s.d. (**P < 0.01). **b** CD31 IHC staining was performed in normal (N) and glioma (G) tissues we collected. *Scale bars* represent 100 μm (*upper*) and 200 μm (*lower*). **c** Amount of capillary-like tubes generated by the HUVEC miR-9 mimic/NC cells was counted under a microscope 48 h post transfection. *Scale bars* represent 200 μm. Data are shown as the mean ± s.d. (**P < 0.01; *n* = 3 independent experiments). **d** Number and length of the novel sprouts derived from HUVEC miR-9 mimic/NC and HUVEC miR-9 inhibitor/NC cells were examined under a microscope. *Scale bars* represent 100 μm. Data are represented as the mean ± s.d. (**P < 0.01 and ***P < 0.001; *n* = 3 independent experiments). **e** Cell adhesive force assay was utilized to evaluate the adhesion ability of HUVEC miR-9 mimic/NC cells at 0.5, 1, 2 and 4 h. Data are shown as the mean ± s.d. (*P < 0.05, **P < 0.01 and ***P < 0.001; *n* = 3 independent experiments). *Scale bars* represent 500 μm. **f** Migration and invasion of the HUVEC miR-9 mimic/NC cells was determined through non-coated (*upper*) and Matrigel-coated (*lower*) transwell assays. Data are shown as the mean ± s.d. (**P < 0.01; *n* = 3 independent experiments). *Scale bars* represent 100 μm

In addition to the aberrant expression of miR-9 in glioma tissues, data from GSE93850 also showed a higher level of miR-9 in glioma patient serum (Fig. 2a), indicating that in addition to its role in glioma cells themselves, miR-9 might also play a role on distant cells. Consistent with this observation, we found that with an increase of miR-9 expression, the microvessel density in the glioma tissues became larger, suggesting a possible correlation between miR-9 and angiogenesis (Fig. 2b). We thus attempted to investigate the direct effects of miR-9 on angiogenesis. Our data showed that miR-9 overexpression in HUVECs had no influence on apoptosis and cell cycle (Additional file 2: Figure S3a-S3c). However, more capillary-like tubes were formed when miR-9 mimics were transfected in HUVECs (Fig. 2c). Additionally, the increase in miR-9 induced HUVECs to generate more and longer sprouts, whereas inhibiting miR-9 expression markedly suppressed the sprout formation and sprout length (Fig. 2d). Meanwhile, cell adhesive force assay suggested that overexpression of miR-9 in HUVECs significantly increased the plate-adhesive cell number compared with the NC group (Fig. 2e). Furthermore, increased miR-9 in HUVECs was correlated with a marked elevation in the cell migration and invasion in transwell assay (Fig. 2f) as well as wound-healing assay (Additional file 2: Figure S3d). In summary, these data indicated that miR-9 was an activator in strengthening the angiogenesis features of the HUVECs to help achieve vascularization.

### MiR-9 is secreted from glioma cells via exosomes and induces neovascularization

**Fig. 3:**
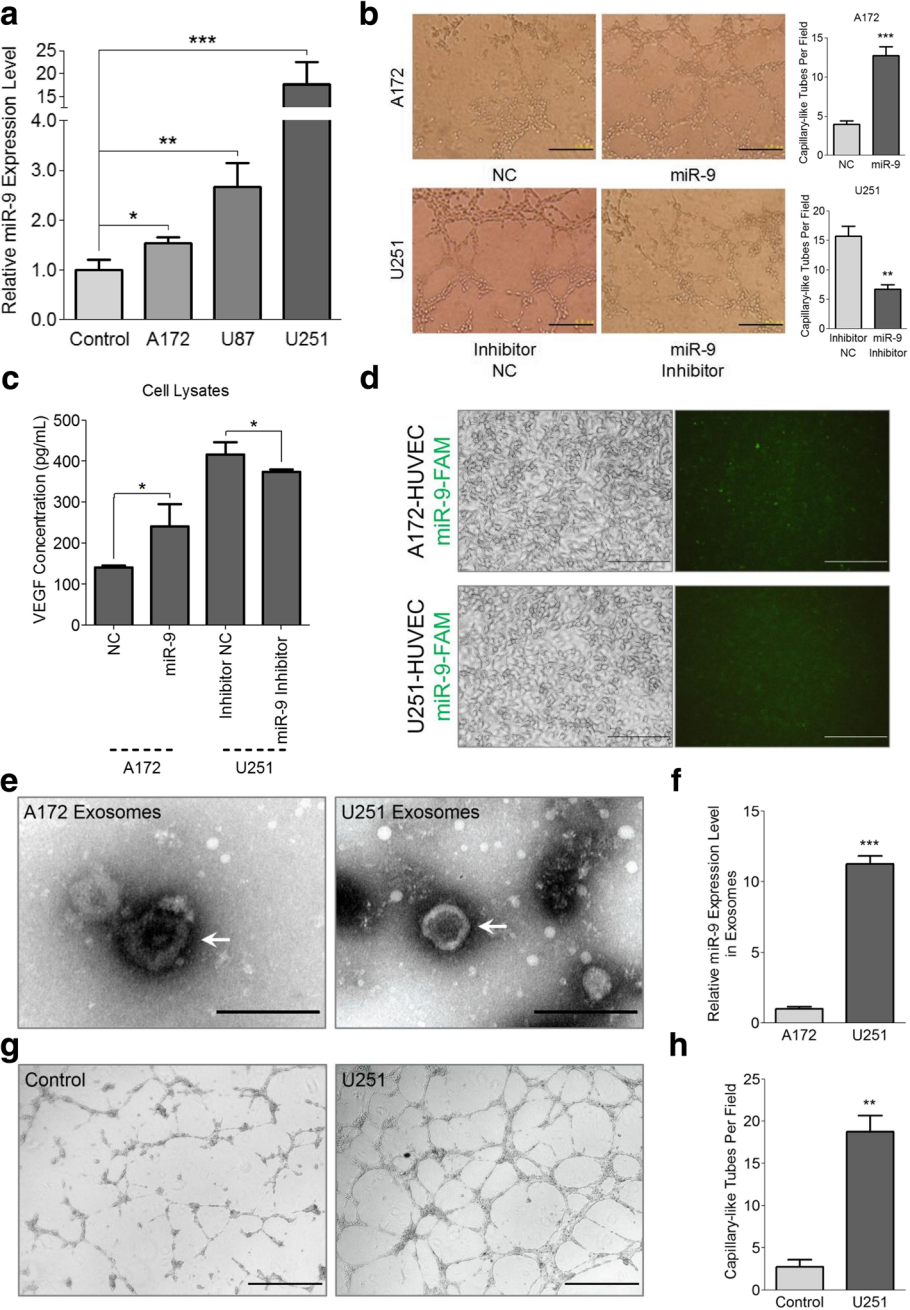
Secreted miR-9 derived from glioma cells enhances angiogenesis in HUVECs. **a** The HUVECs were cultured alone or co-cultured with A172, U87 and U251 cells, respectively. The expression of miR-9 in HUVECs was detected via qRT-PCR after co-culture. Data are presented as the mean ± s.d. (*P < 0.05, **P < 0.01 and ***P < 0.001; *n* = 3 independent experiments). **b** HUVECs were cultured with the conditional medium from A172 miR-9 mimic/NC and U251 miR-9 inhibitor/NC cells, and the amount of capillary-like tubes was determined after 48 h. Photos were taken and the tubes were counted under a microscope at the indicated time. *Scale bars* represent 200 μm. Data are represented as the mean ± s.d. (**P < 0.01 and ***P < 0.001; *n* = 3 independent experiments). **c** Expression level of VEGF in the cell lysates of the A172 miR-9 mimic/NC and U251 miR-9 inhibitor/NC cells were analyzed by ELISA assays. Error bars represent the s.d. (*P < 0.05; *n* = 3 independent experiments). **d** The HUVECs cultured via the conditional medium from A172 miR-9-FAM and U251 miR-9-FAM cells were observed by a fluorescence microscope. *Scale bars* represent 100 μm. **e** The A172 and U251 exosomes were observed and taken photos under a transmission electron microscope. Exosomes are marked by the *white arrows*. *Scale bars* represent 200 nm. **f** MiR-9 levels within the A172 and U251 exosomes were assessed by qRT-PCR analysis. Data are shown as the mean ± s.d. (***P < 0.001; *n* = 3 independent experiments). **g** and **h** Capillary-like tubes from the U251 exosomes or control treated HUVECs were taken photos (**g**) and counted (**h**) under a microscope. *Scale bars* represent 500 μm. Data are represented as the mean ± s.d. (**P < 0.01; *n* = 3 independent experiments)

Based on the existing results, we speculated that miR-9 is likely to be secreted from the glioma cells and absorbed by the HUVECs, thus initiating the glioma-related neovascularization. Hence, we performed a series of assays to confirm this hypothesis. First, a co-culture system was introduced to explore whether glioma cells can secrete miR-9. As shown in Fig. 3a, endogenous miR-9 expression level in cultured HUVECs was relatively low, but when co-cultured with glioma cells (A172, U87 and U251) for 72 h, the expression levels of miR-9 in HUVECs were markedly increased, especially in the cells co-cultured with the U251 cells whose endogenous miR-9 level was the highest. Besides, the expression of miR-9 in HUVECs increased in a time-dependent manner when we used conditional medium that harvested at different time (Additional file 2: Figure S4a). Additionally, we found that incubation with miR-9 mimic conditional medium significantly enhanced the tube formation ability of the HUVECs, while miR-9 inhibitor conditional medium dramatically reduced the amount of novel capillary-like tubes (Fig. 3b). Meanwhile, VEGF was significantly up-regulated in the cell lysates from the miR-9 mimic transfected A172 cells and down-regulated in those from miR-9 inhibitor transfected U251 cells (Fig. 3c). On the contrary, the expression levels of endostatin were significantly decreased when miR-9 was overexpressed in A172 cells and markedly increased when miR-9 was knocked down in U251 cells in both conditional medium and cell lysates (Additional file 2: Figure S4b and S4c), indicating that the pro-angiogenesis elements were in a dominant state under the conditions of miR-9 expression.

Next, we introduced FAM-labeled miR-9 into the co-culture system to track miR-9 transportation. Twenty-four hours after miR-9-FAM transfection, A172 and U251 cells were marked by “green” signals, indicating that the exogenous miR-9 had been absorbed by cells (Additional file 2: Figure S4d). Transfection supernatant was discarded and fresh medium was re-added to cultivate the A172 miR-9-FAM and U251 miR-9-FAM cells for another 72 h. Then, cell supernatant was collected as conditional medium and was used to culture the HUVECs for 24 h. Results showed a number of the HUVECs had turned “green” after the 24 h cultivation (Fig. 3d), further confirming that miR-9 was secreted from glioma cells and was then taken in by the HUVECs.

Since exosomes play important roles in small RNA transportation, we speculated that miR-9 might be secreted by the glioma cells in the form of exosomes. To test this hypothesis, we purified the exosomes from A172 and U251 cells (Fig. 3e). After extracting the total endogenous RNAs in the A172 and U251 exosomes, we examined miR-9 expression and found the level of miR-9 in the exosomes from U251 cells was probably 10-fold higher than that from A172 cells (Fig. 3f). In addition, when we treated the HUVEC cells with purified exosomes from U251 cells, more capillary-like tubes could be observed (Fig. 3g and h), indicating that it was the functional miR-9 in the secreted exosomes that mediated the angiogenic functions of the conditional medium harvested from glioma cell supernatant.

### MiR-9 induces glioma tumorigenesis and angiogenesis in vivo

**Fig. 4:**
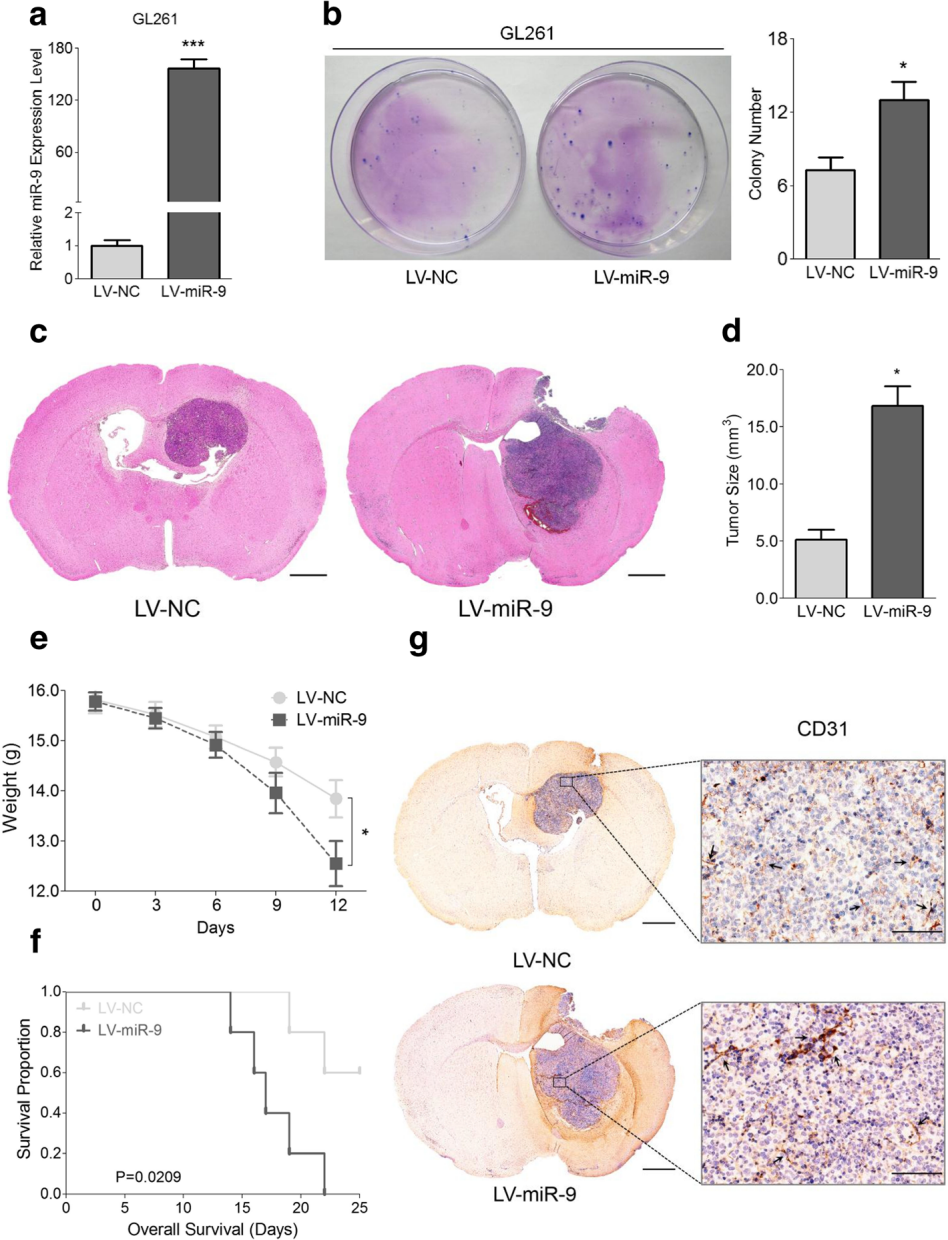
MiR-9 induces glioma tumorigenesis and angiogenesis in vitro and in vivo. **a** Expression level of miR-9 in GL261 LV-miR-9 and GL261 LV-NC cells was detected by qRT-PCR. Data are shown as the mean ± s.d. (***P < 0.001; *n* = 3 independent experiments). **b** Proliferation ability of GL261 LV-miR-9 and GL261 LV-NC cells was measured via the colony formation assay. Data are presented as the mean ± s.d. (*P < 0.05; *n* = 3 independent experiments). **c** Brain sections of the mice from intracranial glioma model were used to perform the H&E staining and representative graphs were shown. *Scale bars* represent 1 mm. **d** Quantification of the tumor size. The tumor size was calculated according to the formula: (W^2^ x L) / 2, W < L. Data are shown as the mean ± s.d. (*P < 0.05; *n* = 5 independent experiments). **e** Body weights of mice in the intracranial glioma model were measured in the 0, 3, 6, 9, 12 days after injection. Data are represented as the mean ± s.d. (*P < 0.05; *n* = 5 independent experiments). **f** Overall survival of the experimental mice was analyzed by the Kaplan-Meier survival curve. Statistical significance was measured through *P* log-rank test. **g** Brain sections of the mice from the intracranial glioma model were used to perform the CD31 IHC staining and representative graphs were shown. *Scale bars* represent 1 mm (*left*) and 50 μm (*right*)

To further investigate the intrinsic role of miR-9 in the glioma tumorigenesis and angiogenesis, we continued to focus on its functions in vivo. We first generated stable miR-9-overexpressing murine GL261 cells by lentiviral infection. As shown in Fig. 4a, expression level of miR-9 was notably increased in GL261 LV-miR-9 cells compared with LV-NC cells. Next, we explored the effects of miR-9 on GL261 LV-miR-9/NC cells before in vivo assays. Consistent with our previous results, miR-9 significantly promoted novel colony formation (Fig. 4b) and accelerated cell cycle progression but had no influence on apoptosis (Additional file 2: Figure S5a). These data provided evidence to support the onco-miR role of miR-9 in glioma and fulfilled preconditions for in vivo experiments.

We then performed an intracranial glioma model to explore the influences of miR-9 on tumor cells in vivo. Mice were sacrificed 2 weeks post cell injection and their brains were taken out and H&E staining were performed. We found that the neoplasms in the GL261 LV-miR-9 group were markedly larger than those in the GL261 LV-NC group (Fig. 4c and d). Meanwhile, mice treated with GL261 LV-miR-9 cells displayed a faster decline in their body weights (Fig. 4e) and a poorer survival rate (Fig. 4f). Furthermore, through CD31 IHC staining, more microvessels could be observed in the tissues from the GL261 LV-miR-9 group than those from the GL261 LV-NC group (Fig. 4g). Similar results were also observed in the subcutaneous glioma model (Additional file 2: Figure S5b-S5e). Collectively, these data further revealed that miR-9 could promote tumorigenesis and angiogenesis during the glioma development and progression in vivo.

### MiR-9 is an endogenous inhibitor of COL18A1, THBS2, PTCH1 and PHD3 in human glioma cells

**Fig. 5:**
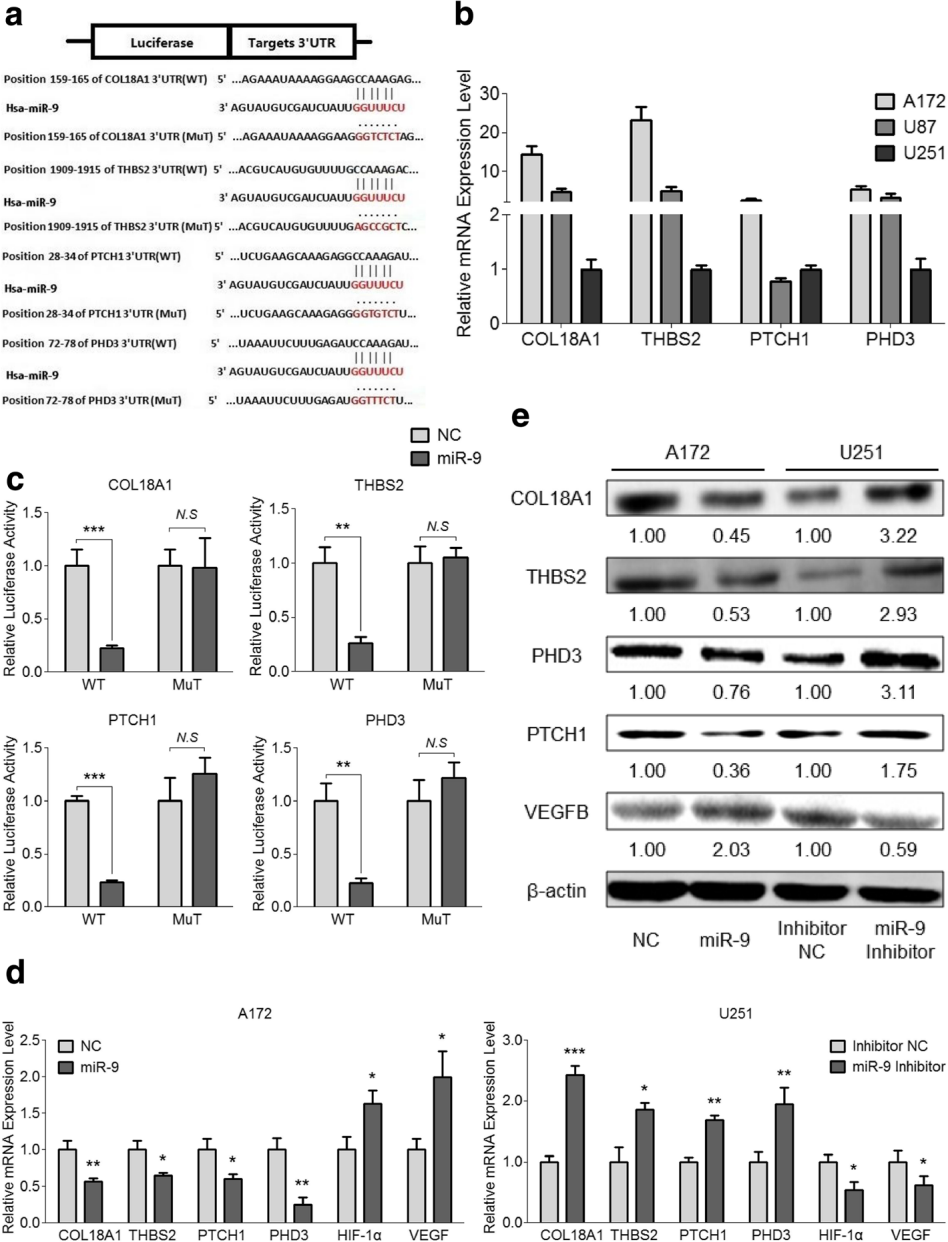
COL18A1, THBS2, PTCH1 and PHD3 are the direct downstream biotargets of miR-9. **a** Diagram of the interaction between the miR-9 (seed site) and target mRNAs (3’-UTRs). The wild-type and mutant sequences of 3’-UTRs are listed. **b** Endogenous levels of COL18A1, THBS2, PTCH1 and PHD3 in glioma cell lines (A172, U87 and U251) were detected by qRT-PCR, respectively. Data are shown as the mean ± s.d.. **c** Dual-luciferase reporter assays were used to measure the relative luciferase activity of the wild-type (WT) or mutant (MuT) reporters for COL18A1, THBS2, PTCH1 and PHD3 when co-transfected with the miR-9 mimic. Data are shown as the mean ± s.d. (*N.S*, no significance; **P < 0.01 and ***P < 0.001; *n* = 3 independent experiments). **d** and **e** Expression of potential targets regulated by miR-9 in A172 miR-9 mimic/NC and U251 miR-9 inhibitor/NC cells was determined by qRT-PCR (**d**) and western blot analysis (**e**). Error bars represent the s.d. (*P < 0.05, **P < 0.01 and ***P < 0.001; *n* = 3 independent experiments)

As our data show, miR-9 is essential for the malignant properties of glioma cells and for the vascular formation during the glioma development. However, mechanisms underlying these phenomena are still unclear. Therefore, we first performed a bioinformatics study and found four candidates, collagen type XVIII alpha 1 chain (COL18A1), thrombospondin 2 (THBS2), patched 1 (PTCH1) and egl-9 family hypoxia inducible factor 3 (PHD3), whose mRNA 3’-UTR contains the miR-9 interactive sequence (Fig. 5a). Next, we determined the endogenous expression levels of these four genes in glioma cell lines (A172, U87 and U251). As shown in Fig. 5b, endogenous levels of these targets displayed a significantly negative correlation with miR-9 expression in glioma cells, strongly indicating an inverse relationship between miR-9 and COL18A1, THBS2, PTCH1 and PHD3.

To further clarify that COL18A1, THBS2, PTCH1 and PHD3 are direct targets of miR-9, we amplified corresponding wild-type (WT) and mutant (MuT) 3’-UTRs and cloned them into the PGL3 luciferase reporter vectors. Relative luciferase activity of wild-type (WT) vectors was dramatically diminished upon co-transfection with miR-9 mimic. However, luciferase activity of the vectors harboring mutant (MuT) 3’-UTRs of these genes remained unchanged (Fig. 5c). Additionally, overexpression of miR-9 significantly reduced the expression of COL18A1, THBS2, PTCH1 and PHD3, while knockdown of miR-9 markedly increased the expression of these four targets at both the mRNA (Fig. 5d) and protein (Fig. 5e) levels. These results support bioinformatics prediction and confirm that COL18A1, THBS2, PTCH1 and PHD3 are indeed direct targets of miR-9 in the glioma cells.

Since PHD3 is the downstream target of miR-9, we speculated that the canonical HIF-1α/VEGF signaling pathway is involved in regulating glioma angiogenesis. Data showed that up-regulating miR-9 could promote HIF-1α and VEGF expression, while HIF-1α and VEGF were inhibited with the presence of miR-9 inhibitor compared with the corresponding NC (Fig. 5d). Additionally, similar alterations in VEGFB were also observed when miR-9 was overexpressed in A172 cells and knocked down in U251 cells (Fig. 5e), which was consistent with the cell lysate ELISA results for these two modified cell lines (Fig. 3c). Therefore, the PHD3-mediated HIF-1α/VEGF signaling pathway is engaged in the regulation of glioma tumorigenesis and is particularly vital for tumor neovascularization.

### MYC/OCT4 dominantly facilitates the miR-9-2 transcript expression by directly binding to its promoter region

**Fig. 6:**
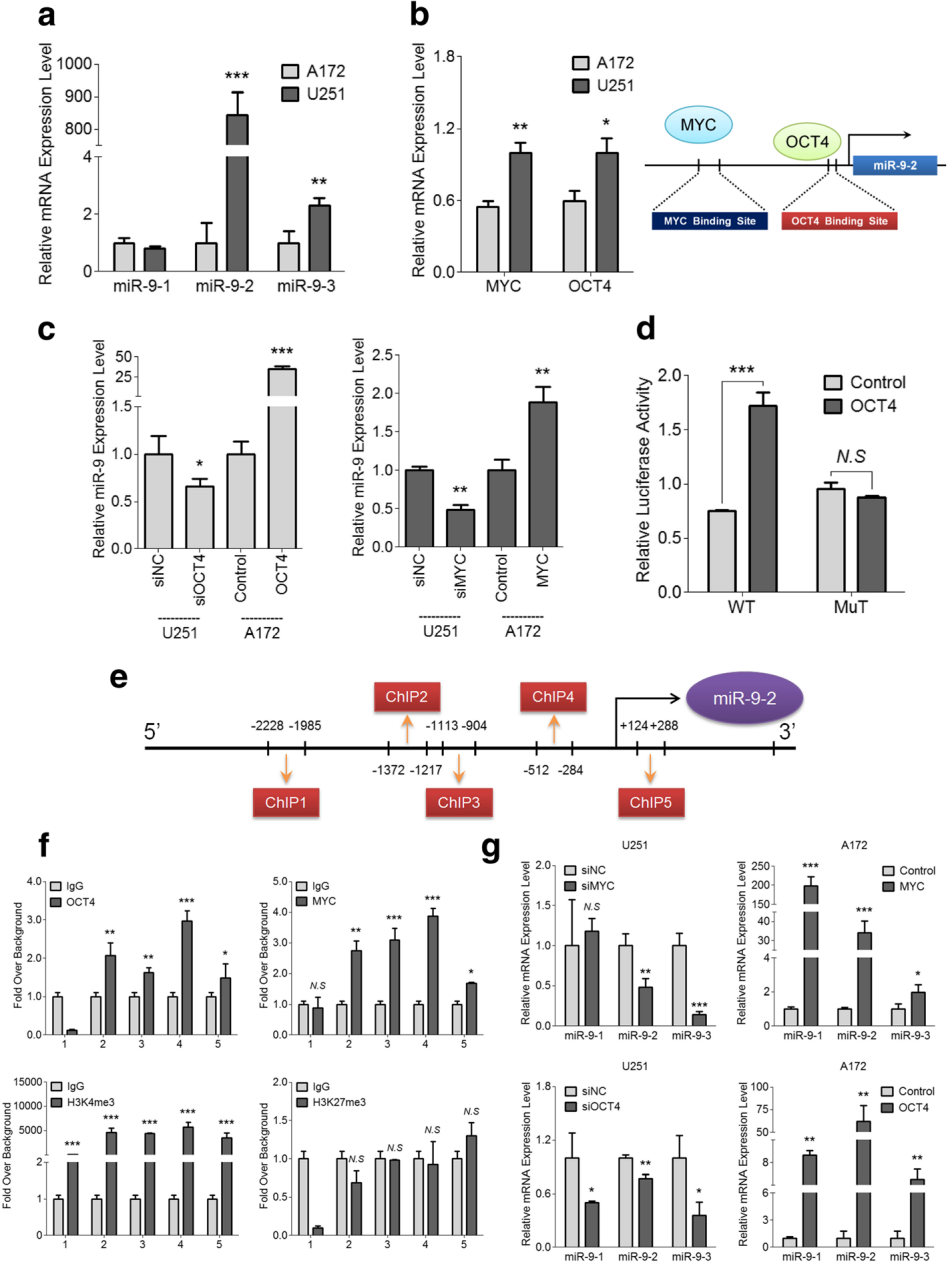
MiR-9 is directly activated by MYC and OCT4 in glioma cells. **a** Endogenous expression levels of the three miR-9 transcripts (miR-9-1, miR-9-2 and miR-9-3) were examined in A172 and U251 cells by qRT-PCR analysis. Data are shown as the mean ± s.d. (**P < 0.01 and ***P < 0.001; *n* = 3 independent experiments). **b** Endogenous expression levels of MYC and OCT4 were detected by qRT-PCR in A172 and U251 cells (*left*), and the model diagram exhibits the predicted MYC/OCT4 binding site on the miR-9-2 promoter region (*right*). Data are presented as the mean ± s.d. (*P < 0.05 and **P < 0.01; *n* = 3 independent experiments). **c** qRT-PCR analysis was applied to determine the miR-9 expression in A172 MYC/OCT4 and control cells and in U251 MYC/OCT4 siRNA and siNC cells. Error bars represent the s.d. (*P < 0.05, **P < 0.01 and ***P < 0.001; *n* = 3 independent experiments). **d** Dual-luciferase reporter assays were used to assess the relative luciferase activity of vectors with the wild-type or mutant OCT4 binding site upon co-transfection with an OCT4-expressing vector or control. Data are presented as the mean ± s.d. (*N.S*, no significance; ***P < 0.001; *n* = 3 independent experiments). **e** Diagram of the location and amplified fragments of the five ChIP primers on the promoter region of miR-9-2. **f** Enrichment of MYC, OCT4, H3K4me3 and H3K27me3 at the miR-9-2 promoter region was tested by ChIP assay in the U251 cells. Data are shown as the mean ± s.d. (*N.S*, no significance; *P < 0.05, **P < 0.01 and ***P < 0.001; *n* = 3 independent experiments). **g** Expression levels of the three miR-9 transcripts in the A172 MYC/OCT4 and control cells and in the U251 MYC/OCT4 siRNA and siNC cells. Error bars represent the s.d. (*N.S*, no significance; *P < 0.05, **P < 0.01 and ***P < 0.001; *n* = 3 independent experiments)

Since miR-9 functions as an onco-miR in glioma, we hypothesized that miR-9 is likely to be activated by some pro-tumor factors. In fact, mature miR-9 consists of 3 independent transcripts with different locations: miR-9-1 (1q22), miR-9-2 (5q14.3) and miR-9-3 (15q26). Hence, we first examined the endogenous expressions of these three transcripts in A172 and U251 cells. As shown in Fig. 6a, miR-9-1 and miR-9-3 were nearly equal in these two cell lines, while miR-9-2 was notably higher in U251 cells than in A172 cells, indicating that total miR-9 expression in glioma cells, at least in these two cell lines, is dominantly influenced by miR-9-2 as opposed to miR-9-1 or miR-9-3.

Next, we focused on the potential regulators that initiate the transcription process of miR-9-2 via the bioinformatics prediction. Intriguingly, we found that the promoter region of miR-9-2 contained MYC and OCT4 binding sites. Therefore, we continued to verify the potential regulatory relationship between miR-9-2 and MYC/OCT4. We first detected the endogenous expressions of MYC and OCT4 in A172 and U251 cells. Results showed that both of MYC and OCT4 were higher in U251 cells than in A172 cells (Fig. 6b). Meanwhile, overexpression of MYC and OCT4 could significantly increase miR-9 level, while transfection of specific small interfering RNAs targeting MYC or OCT4 markedly inhibited miR-9 expression (Additional file 2: Figure S5f and Fig. 6c). These results suggest a positive correlation between miR-9 and MYC/OCT4 in glioma cells.

To further identify the regulatory relationship between MYC/OCT4 and miR-9, we cloned the predicted wild-type (WT) and mutant (MuT) OCT4 binding sites into the PGL3 luciferase reporter vector. The luciferase activity of wild-type (WT) reporter was significantly increased upon co-transfection with an OCT4-overexpressing vector, whereas the mutant (MuT) reporter showed no marked alterations (Fig. 6d). Next, we designed five ChIP primers to amplify the miR-9-2 promoter region (− 2500 bp ~ + 500 bp) and performed a ChIP assay in U251 cells that contained the highest endogenous miR-9 among the studied glioma cells (Fig. 6e). As shown in Fig. 6f, the miR-9-2 promoter region was significantly enriched with MYC and OCT4 compared with IgG, indicating that these two transcription factors can directly bind to the promoter region of miR-9-2 and launch its transcription. Additionally, the pro-transcription biomarker H3K4me3 was prominently recruited, whereas the suppressive biomarker H3K27me3 remained undetectable on the miR-9-2 promoter; together, these findings explain the high miR-9-2 level in U251 cells.

We further explored the correlation between MYC/OCT4 and miR-9 transcript expression in glioma cells. Results showed that levels of the three miR-9 transcripts paralleled the changes in MYC or OCT4. Of note, miR-9-2 was the key downstream target of MYC or OCT4, as its level, not that of miR-9-1 or miR-9-3, was markedly influenced (Fig. 6g). Collectively, these results indicate that the aberrant activation of miR-9, especially of miR-9-2, in glioma cells is due to the enrichment of MYC/OCT4 on its promoter region. Therefore, inhibiting or blocking this binding may be a novel strategy for glioma therapy.

## Discussion

Recently, multiple elements, including the interleukin (IL) and Notch signaling pathways, and miRNAs have been implicated in glioma tumorigenesis [20]. Despite the remarkable endeavors in developing treatments to improve patient prognosis, total survival rate of patients with malignant glioma remains low level [21], and the lack of knowledge regarding the mechanisms of glioma tumorigenesis has not been mitigated. There is an urgent requirement to identify the sensitive and effective biomarkers for glioma diagnostics and therapeutics. In present study, we attempted to characterize the relationship between miR-9 and glioma. We confirmed that miR-9 is a key promoter for glioma tumorigenesis and angiogenesis, thus providing results to better understand the molecular mechanism of glioma and to identify an effective target for its future therapy.

Expression pattern of miR-9 in tumors is closely related to the tissue source and distribution. In human cervical [22], ovarian [23] and prostate [24] cancers, miR-9 is positively correlated with malignant phenotypes and an unfavorable patient prognosis. Nevertheless, miR-9 down-regulation is considered as a trigger for the progression of non-small cell lung cancer [25] and gastric cancer [26]. Furthermore, role of miR-9 in CNS neoplasms is still controversial. Zhang H [27] reported that miR-9 abolished the malignant phenotypes of neuroblastoma cells via targeting matrix metalloproteinase 14 (MMP-14). In another report, Gomez GG [28] found the EGFR mutation-induced miR-9 suppression could elevate glioblastoma tumorigenicity by activating FOXP1. On the contrary, Schraivogel D [29] suggested that miR-9 was highly abundant and negatively regulated tumor suppressor CAMTA1 in the glioblastoma stem cells. Wu Z [30] proposed a negative association between miR-9 and outcomes of glioma patients. In our study, we discovered that miR-9 was notably up-regulated in glioma cells and primary tumor tissues compared with normal tissues. In addition, miR-9 significantly stimulated the glioma cell proliferation, migration and invasion, and promoted the generation of novel blood vessels in vitro and in vivo, further suggesting that miR-9, which contains tissue heterogeneity, is an intrinsic onco-miR in human glioma.

The four verified targets of miR-9 in our study are closely correlated with the malignant characteristics of glioma cells. Human collagen XVIII protein, encoded by single COL18A1 gene, is produced in three tissue-specific isoforms that differ from the size and N-terminal non-collagenous sequences but harbor common collagenous and C-terminal non-collagenous domains [31]. Of note, endostatin resides at the end of the C-terminal non-collagenous domain in each collagen XVIII isoform and is formed from the process of proteolytic cleavage with specific enzymes, for example the matrix metalloproteases (MMP), elastase and cathepsins, leading to its release from parental collagen XVIII [32]. As a canonical angiogenesis inhibitor, endostatin exerts its functions by suppressing the proliferation and migration along with inducing apoptosis of endothelial cells [33–35], deploying diverse receptors [36, 37], triggering multiple signaling pathways to mediate the autophagy in the endothelial cells [38] and blocking the activation of MMPs [39]. In addition to COL18A1, THBS2 is involved in the focal adhesion pathway in human SNB19 glioma cells [40] and functions as an endogenous inhibitor of angiogenesis [41]. As a vital negative component of the Sonic Hedgehog (SHH) signaling pathway, PTCH1 participates in the suppression of glioma growth [42] and its inhibition by miR-9 can induce tumor growth both in our study and in a previous report [43]. Finally, PHD3 stabilizes HIF-1α and serves as a pivotal regulator of HIF-1α-mediated angiogenesis [44]. In our study, we confirm that miR-9 can directly bind to the 3’-UTR of COL18A1, THBS2, PTCH1 and PHD3, promote the degradation of these mRNAs and initiate HIF-1α/VEGF signal transduction, thus markedly enhancing tumorigenesis and angiogenesis during the glioma progression.

MYC and OCT4 are two canonical stem cell factors which are implicated in the development and progression of various solid tumors, especially glioma. Enrichment of MYC on the EGFR/EGFRvIII promoter region can notably facilitate the malignant phenotypes of glioma cells [45]. OCT4 is found to be a favorable element for driving glioma recurrence [46]. In addition, these two transcription factors also participate in novel vasculature formation. Recruitment of MYC to the angiopoietin-like 4 (Angptl4) promoter region stimulates its expression and enhances angiogenesis [47]; Aberrant expression of OCT4 in the HUVECs can remodel endothelial cells into endothelial progenitor cells (EPCs), which effectively enhances their angiogenesis potential [48]. Moreover, MYC and OCT4 are closely correlated with the expression and function of multiple miRNAs [49, 50]. In this study, we elucidate that MYC or OCT4 increases the expression of miR-9 by directly binding to its promoter region and thus regulates glioma tumorigenesis and angiogenesis through a miR-9 involved axis.

## Conclusions

In summary, our results suggest that miR-9 is frequently up-regulated in human glioma tissues and cells and functions as an onco-miR to enhance cell proliferation, migration, invasion and angiogenesis during the development and progression of glioma in vitro and in vivo. Mechanistically, mature miR-9 recognizes and binds to the 3’-UTRs of COL18A1, THBS2, PTCH1 and PHD3 to degrade these mRNAs, leading to a dysfunction of HIF-1α/VEGF signaling pathway and malignant phenotypes. In addition, maintenance of miR-9 in glioma cells depends on direct activation by MYC or OCT4 on the miR-9 promoter (Additional file 2: Figure S6). Therefore, we provide an insight into the regulation of glioma and offer a novel target for the diagnosis and treatment of glioma patients.

## Additional files

Additional file 1: **Table S1.** Information on the 18 tissues of glioma patients collected for clinical study. **Table S2.** Information on the oligonucleotide sequences in this study. **Table S3.** Information on the primer sequences for qRT-PCR analysis in this study. **Table S4.** Information on the antibodies used in this study. **Table S5.** Information on the PCR primers for luciferase reporter assay in this study. **Table S6.** Information on the primer sequences for ChIP assay in this study. (ZIP 450 kb)

Additional file 2: **Figure S1.** MiR-9 is aberrantly expressed in the glioma specimens and shows tissue-dependent distribution. **Figure S2.** Knockdown of miR-9 suppresses malignant phenotypes of glioma cells. **Figure S3.** MiR-9 is involved in the regulation of basic biological behaviors of the HUVECs. **Figure S4.** MiR-9 acts as an angiogenesis inducer that is secreted from glioma cells and taken in by the HUVECs. **Figure S5.** MiR-9 promotes the glioma growth and novel vessel formation in vivo. **Figure S6.** Pattern diagram that summarize the regulatory model in our study. (PDF 990 kb)

## Abbreviations

CNS: Central nervous system
COL18A1: Collagen type XVIII alpha 1 chain
HUVEC: Human umbilical vein endothelial cell
MYC: v-Myc avian myelocytomatosis viral oncogene homolog
OCT4: POU class 5 homeobox 1
PHD3: egl-9 family hypoxia inducible factor 3
PTCH1: Patched 1
THBS2: Thrombospondin 2
VEGF: Vascular endothelial growth factor
