# Supplementary material for: MiR-9 promotes tumorigenesis and angiogenesis and is activated by MYC and OCT4 in human glioma
Source: J Exp Clin Cancer Res. 2019 Feb 22;38:99. doi: 10.1186/s13046-019-1078-2 (PMC6385476; doi:10.1186/s13046-019-1078-2)
Supplement: Supplementary file 1 — Table S1. Information on the 18 tissues of glioma patients collected for clinical study. Table S2. Information on the oligonucleotide sequences in this study. Table S3. Information on the primer sequences for qRT-PCR analysis in this study. Table S4. Information on the antibodies used in this study. Table S5. Information on the PCR primers for luciferase reporter assay in this study. Table S6. Information on the primer sequences for ChIP assay in this study. (ZIP 450 kb) [file 13046_2019_1078_MOESM1_ESM.zip › Table S1.pdf]

Supplementary Information

Table S1

Information on the 18 tissues of glioma patients collected for clinical study

**Patient Number**	**Gender**	**Age**	**Pathological Diagnosis**	**WHO Grade**
1	Female	47	Diffuse Astrocytoma	II
2	Female	60	Anaplastic Astrocytoma	III
3	Male	40	Glioblastoma Multiforme	IV
4	Male	45	Glioblastoma Multiforme	IV
5	Male	53	Glioblastoma Multiforme	IV
6	Male	57	Oligodendroglioma	II
7	Female	55	Glioblastoma Multiforme	IV
8	Female	41	Oligodendroglioma	II
9	Male	38	Oligodendroglioma	II
10	Male	40	Ganglioglioma	II
11	Male	30	Glioblastoma Multiforme	IV
12	Male	48	Anaplastic Astrocytoma	III
13	Male	50	Anaplastic Oligodendroglioma	III
14	Female	54	Anaplastic Astrocytoma	III
15	Female	47	Oligodendroglioma	II
16	Female	29	Glioblastoma Multiforme	IV
17	Male	52	Glioblastoma Multiforme	IV
18	Female	44	Oligodendroglioma	II
