# Supplementary material for: MiR-9 promotes tumorigenesis and angiogenesis and is activated by MYC and OCT4 in human glioma
Source: J Exp Clin Cancer Res. 2019 Feb 22;38:99. doi: 10.1186/s13046-019-1078-2 (PMC6385476; doi:10.1186/s13046-019-1078-2)
Supplement: Supplementary file 1 — Table S1. Information on the 18 tissues of glioma patients collected for clinical study. Table S2. Information on the oligonucleotide sequences in this study. Table S3. Information on the primer sequences for qRT-PCR analysis in this study. Table S4. Information on the antibodies used in this study. Table S5. Information on the PCR primers for luciferase reporter assay in this study. Table S6. Information on the primer sequences for ChIP assay in this study. (ZIP 450 kb) [file 13046_2019_1078_MOESM1_ESM.zip › Table S2.pdf]

Supplementary Information

Table S2

Information on the oligonucleotide sequences in this study

Oligonucleotide	Sense (5’-3’)	Anti-sense (5’-3’)
mimic NC/siNC	UUCUCCGAACGUGUCACGUTT	ACGUGACACGUUCGGAGAATT
miR-9 mimic	UCUUUGGUUAUCUAGCUGUAUGA	AUACAGCUAGAUAACCAAAGAUU
inhibitor NC	CAGUACUUUUGUGUAGUACAA	
miR-9 inhibitor	CUACCUGCACGAACAGCACUUUG	
siMYC	AACGUUAGCUUCACCAACAUU	UGUUGGUGAAGCUAACGUUUU
siOCT4	GGAGGAAGCUGACAACAAUTT	AUUGUUGUCAGCUUCCUCCTT
