# Supplementary material for: MiR-9 promotes tumorigenesis and angiogenesis and is activated by MYC and OCT4 in human glioma
Source: J Exp Clin Cancer Res. 2019 Feb 22;38:99. doi: 10.1186/s13046-019-1078-2 (PMC6385476; doi:10.1186/s13046-019-1078-2)
Supplement: Supplementary file 1 — Table S1. Information on the 18 tissues of glioma patients collected for clinical study. Table S2. Information on the oligonucleotide sequences in this study. Table S3. Information on the primer sequences for qRT-PCR analysis in this study. Table S4. Information on the antibodies used in this study. Table S5. Information on the PCR primers for luciferase reporter assay in this study. Table S6. Information on the primer sequences for ChIP assay in this study. (ZIP 450 kb) [file 13046_2019_1078_MOESM1_ESM.zip › Table S3.pdf]

Supplementary Information

Table S3

Information on the primer sequences for qRT-PCR analysis in this study

Gene	Forward (5'-3')	Reverse (5'-3')
miR-9	CGGTCTTTGGTTATCTAGCTGTATG	RR716, SYBR® PrimeScript™ miRNA RT-PCR Kit, TaKaRa
miR-9-1	CAGAGAAGGGCAGTGGAGAC	ACGACAGAGACCGAAAAAGG
miR-9-2	CTAACGCTGCCGGAGATTAC	TACTTGCCGCGCTTAAGATT
miR-9-3	GCGCTCGAGGCTCTCTAAG	GAGGGGATGGACAGACACAC
COL18A1	GCCATACTTTCCTGTATAGTTC	AGGTGAATGTCAGCATCA
THBS2	GATTAGAAGACAACAGGCATAG	GTAGGACAAGAGGAGAGTTC
PTCH1	AAGTTGGAGGACGAGTAA	GACATTAGCACCTTCTTCTT
PHD3	TTCTCAACGACTGCTCTATT	CCTGTCTTCATTGTGCTTAC
HIF-1α	CCGAGGAAGAACTATGAA	GTTGGTTACTGTTGGTATC
VEGF	AGCCTTGCCTTGCTGCTCTA	GTGCTGGCCTTGGTGAGG
MYC	CTCCTGGCAAAAGGTCAGAG	TCGGTTGTTGCTGATCTGTC
OCT4	GTATTCAGCCAAACGACCATCT	GCTTCCTCCACCCACTTCT
U6	CTCGCTTCGGCAGCACA	AACGCTTCACGAATTTGCGT
β-actin	TGGCATCCACGAAACTACC	GTGTTGGCGTACAGGTCTT
