# Supplementary material for: MiR-9 promotes tumorigenesis and angiogenesis and is activated by MYC and OCT4 in human glioma
Source: J Exp Clin Cancer Res. 2019 Feb 22;38:99. doi: 10.1186/s13046-019-1078-2 (PMC6385476; doi:10.1186/s13046-019-1078-2)
Supplement: Supplementary file 1 — Table S1. Information on the 18 tissues of glioma patients collected for clinical study. Table S2. Information on the oligonucleotide sequences in this study. Table S3. Information on the primer sequences for qRT-PCR analysis in this study. Table S4. Information on the antibodies used in this study. Table S5. Information on the PCR primers for luciferase reporter assay in this study. Table S6. Information on the primer sequences for ChIP assay in this study. (ZIP 450 kb) [file 13046_2019_1078_MOESM1_ESM.zip › Table S4.pdf]

Supplementary Information

Table S4

Information on the antibodies used in this study

Antigen	Species	Application and Dilution	Source
COL18A1	Mouse	WB (1:500)	Santa Cruz Biotechnology #sc-32720
THBS2	Goat	WB (1:500)	Santa Cruz Biotechnology #sc-12313
PTCH1	Mouse	WB (1:500)	Origene #TA500123
PHD3	Rabbit	WB (1:500)	Sangon Biotech #D121164
VEGFB	Rabbit	WB (1:500)	Sangon Biotech #D151121
CD31	Rabbit	IHC (1:50)	Abcam #ab28364
CD34	Rabbit	IHC (1:50)	Abcam #ab185732
MYC	Rabbit	5μg in ChIP	Cell Signaling Technology #9402
OCT4	Rabbit	5μg in ChIP	Abcam #ab19857
H3K4me3	Rabbit	5μg in ChIP	Cell Signaling Technology #9751
H3K27me3	Rabbit	5μg in ChIP	Cell Signaling Technology #9733
β-actin	Mouse	WB (1:2000)	Sigma-Aldrich #A1978
Anti-rabbit IgG	Goat	WB (1:4000)	Cell Signaling Technology #7074
Anti-mouse IgG	Goat	WB (1:4000)	Jing Cai #PB002H
Anti-goat IgG	Rabbit	WB (1:4000)	Boster #BA1060
