# Supplementary material for: MiR-9 promotes tumorigenesis and angiogenesis and is activated by MYC and OCT4 in human glioma
Source: J Exp Clin Cancer Res. 2019 Feb 22;38:99. doi: 10.1186/s13046-019-1078-2 (PMC6385476; doi:10.1186/s13046-019-1078-2)
Supplement: Supplementary file 1 — Table S1. Information on the 18 tissues of glioma patients collected for clinical study. Table S2. Information on the oligonucleotide sequences in this study. Table S3. Information on the primer sequences for qRT-PCR analysis in this study. Table S4. Information on the antibodies used in this study. Table S5. Information on the PCR primers for luciferase reporter assay in this study. Table S6. Information on the primer sequences for ChIP assay in this study. (ZIP 450 kb) [file 13046_2019_1078_MOESM1_ESM.zip › Table S5.pdf]

Supplementary Information

Table S5

Information on the PCR primers for luciferase reporter assay in this study

Gene	Sense (5'-3')	Anti-sense (5'-3')
**Wild Type**		
COL18A1	CGGAATTCACAGCTTCATGACTGCCT	GCTGCAGAGATGAAGTCAGCACCTG
THBS2	CGGAATTCTTGCGTGTGGAGCTGTAT	GCTGCAGGAGGTTCTAGCTAAGTGC
PTCH1	CGGAATTCGGATTCGAAGGTGGAAGT	GGCTAGCGCCCAATGCACAAATACG
PHD3	CGGATATCACTGAATCTGCCCTCACT	GGCTAGCTTCACTATGACAGCAGCC
OCT4	TTGGTACCATCTTCTCCTTCCCAGCCTAG	TCTCGAGCTGGTTTTTACTGTCTCTCGGC
**Mutation**		
COL18A1	AGGAAGGGTCTCTGTGTA	AATACACAGAGACCCTTCC
THBS2	CGGAATTCTTGCGTGTGGAGCTGTAT	GCTGCAGTTACAGAGCGGCTCAAAAC
PTCH1	CGGAATTCAAGAGGTAGCGTCTTGGA	GGCTAGCGCCCAATGCACAAATACG
PHD3	CGGAATTCTGAGATGGTTTCTTGGCC	GGCTAGCTTCACTATGACAGCAGCC
OCT4	GGTACCTTGCGTGAGAAAGAAGCAATTTAC ATACAGGGAAGAGGCCTGTGAAGGGACGG	TTCTCGAGCCTGACCTTTCTGGTTTTT ACTGTCTCTCGGC
