# Supplementary material for: MiR-9 promotes tumorigenesis and angiogenesis and is activated by MYC and OCT4 in human glioma
Source: J Exp Clin Cancer Res. 2019 Feb 22;38:99. doi: 10.1186/s13046-019-1078-2 (PMC6385476; doi:10.1186/s13046-019-1078-2)
Supplement: Supplementary file 1 — Table S1. Information on the 18 tissues of glioma patients collected for clinical study. Table S2. Information on the oligonucleotide sequences in this study. Table S3. Information on the primer sequences for qRT-PCR analysis in this study. Table S4. Information on the antibodies used in this study. Table S5. Information on the PCR primers for luciferase reporter assay in this study. Table S6. Information on the primer sequences for ChIP assay in this study. (ZIP 450 kb) [file 13046_2019_1078_MOESM1_ESM.zip › Table S6.pdf]

Supplementary Information

Table S6

Information on the primer sequences for ChIP assay in this study

Sets of primer	Forward (5’-3’)	Reverse (5’-3’)
**miR-9-2**		
1	GGGAAAAGTTGGCATCTATGTT	CCCCAACAACTAAAACACTTTTG
2	TGCATTCTAACGCACACACC	TGGCTTCACAGCTCAGTCAT
3	GCAATAGCAAAGTGCCTTCC	TTCCGCTAGCACTACCGACT
4	TGGTGCCTAACCAAAAAGGA	GCAGCAAGCTGTTACGAGTG
5	GCCGAGAGACAGTAAAAACCA	GCAACAACCCCTCTCAAGAC
