# Supplementary material for: MiR-9 promotes tumorigenesis and angiogenesis and is activated by MYC and OCT4 in human glioma
Source: J Exp Clin Cancer Res. 2019 Feb 22;38:99. doi: 10.1186/s13046-019-1078-2 (PMC6385476; doi:10.1186/s13046-019-1078-2)
Supplement: Supplementary file 2 — Figure S1. MiR-9 is aberrantly expressed in the glioma specimens and shows tissue-dependent distribution. Figure S2. Knockdown of miR-9 suppresses malignant phenotypes of glioma cells. Figure S3. MiR-9 is involved in the regulation of basic biological behaviors of the HUVECs. Figure S4. MiR-9 acts as an angiogenesis inducer that is secreted from glioma cells and taken in by the HUVECs. Figure S5. MiR-9 promotes the glioma growth and novel vessel formation in vivo. Figure S6. Pattern diagram that summarize the regulatory model in our study. (PDF 990 kb) [file 13046_2019_1078_MOESM2_ESM.pdf]

Figure S1.

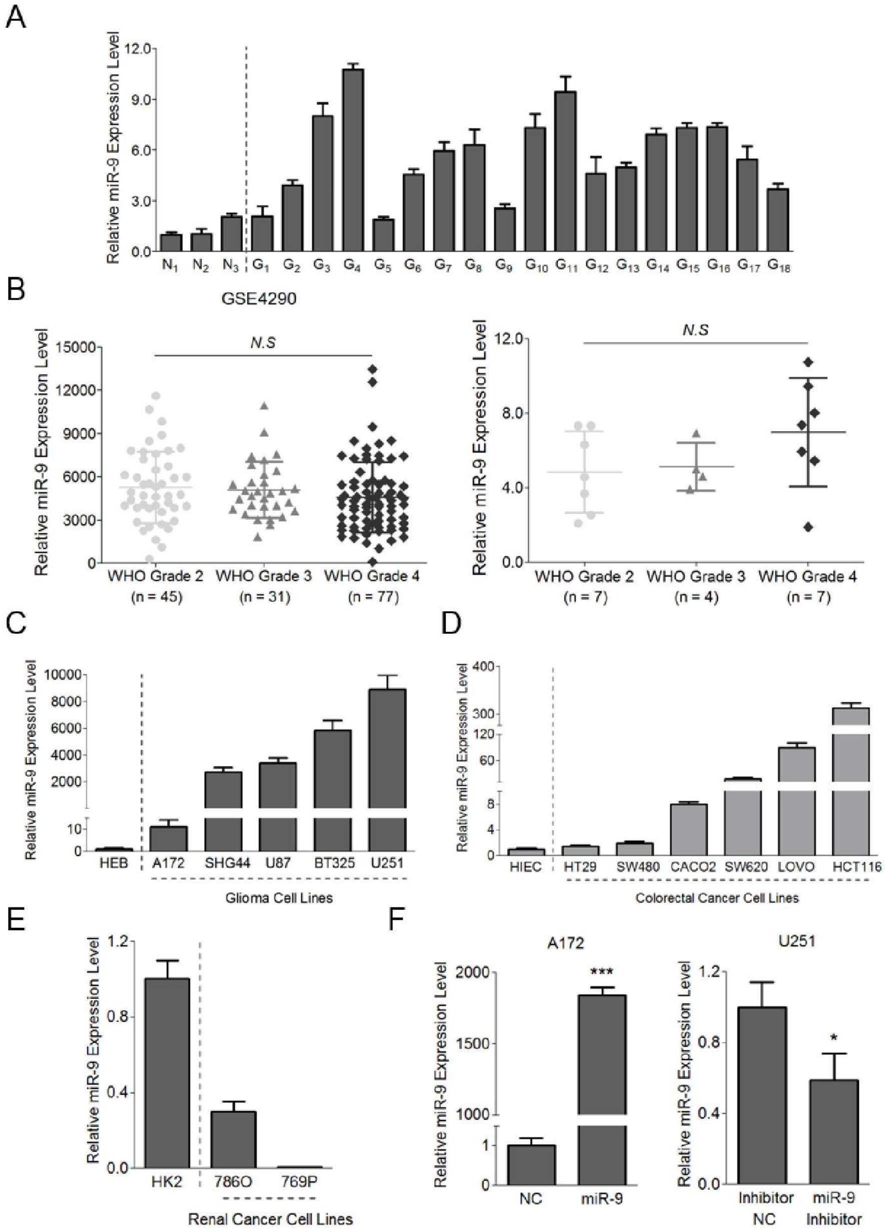

Figure S1. MiR-9 is aberrantly expressed in the glioma specimens and shows tissue-dependent distribution.

(A) qRT-PCR analysis was used to detect the miR-9 expression in the 3 normal (N) and 18 glioma (G) tissues. Data are shown as the mean ± s.d.. Dotted line was applied to differentiate normal tissues from glioma tissues. (B) Scatter diagrams were used to examine the differences between miR-9 and the WHO grade with the data from GSE4290 (left) and the tissues we collected (right). Data are represented as the mean ± s.d.. N.S, no significance. (C) qRT-PCR analysis was used to investigate the endogenous miR-9 level in glioma cells (A172, SHG44, U87, BT325 and U251) and normal glia cells (HEB). Data are presented as the mean ± s.d.. (D and E) Endogenous expression of miR-9 was detected in colorectal (D) and renal (E) normal/cancer cell lines. Data are shown as the mean ± s.d.. (F) Transient transfection efficiency of the miR-9 mimic/NC and miR-9 inhibitor/NC in A172 and U251 cells was examined by qRT-PCR, respectively. Data are presented as the mean ± s.d. (*P < 0.05 and ***P < 0.001; n = 3 independent experiments).

Figure S2.

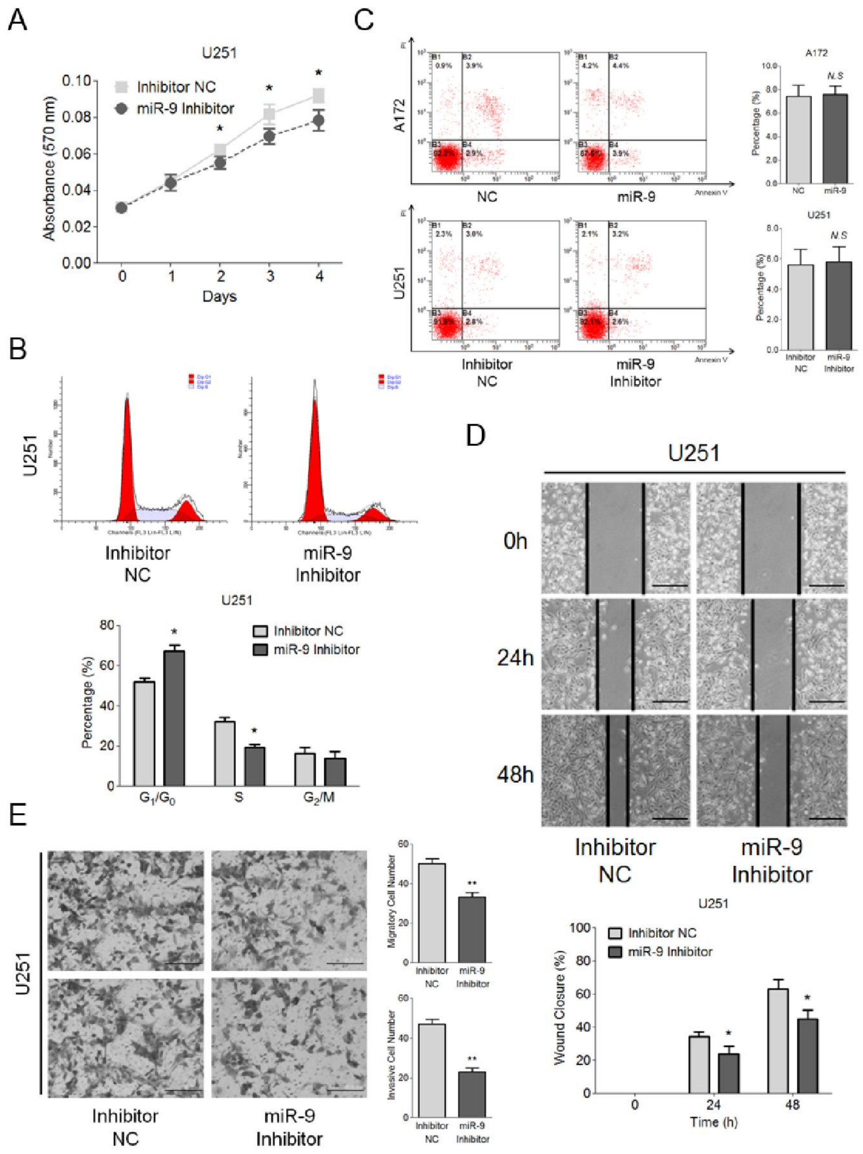

Figure S2. Knockdown of miR-9 suppresses malignant phenotypes of glioma cells.

(A) Proliferation of U251 miR-9 inhibitor/NC cells was measured by MTT assay. Error bars represent the s.d. (*P < 0.05; n = 6 independent experiments). (B) Flow cytometry analysis was applied to evaluate the cell cycle of the U251 miR-9 inhibitor/NC cells. Data are shown as the mean ± s.d. (*P < 0.05; n = 3 independent experiments). (C) Cell apoptosis was analyzed by flow cytometry in the A172 miR-9 mimic/NC and U251 miR-9 inhibitor/NC cells. Data are represented as the mean ± s.d. (N.S, no significance; n = 3 independent experiments). (D) Wound-healing assay was used to evaluate the migration of U251 miR-9 inhibitor/NC cells (upper). Photos were taken at 0, 24 and 48 h, respectively. Histogram was applied for statistical analysis of wound-healing assay for U251 miR-9 inhibitor/NC cells (lower). Scale bars represent 200 µm. Error bars represent the s.d. (*P < 0.05; n = 3 independent experiments). (E) Migration (upper) and invasion (lower) abilities of the U251 miR-9 inhibitor/NC cells were assessed through the non-coated transwell and Matrigel-coated transwell assays, respectively. Scale bars represent 100 µm. Data are presented as the mean ± s.d. (**P < 0.01; n = 3 independent experiments).

Figure S3.

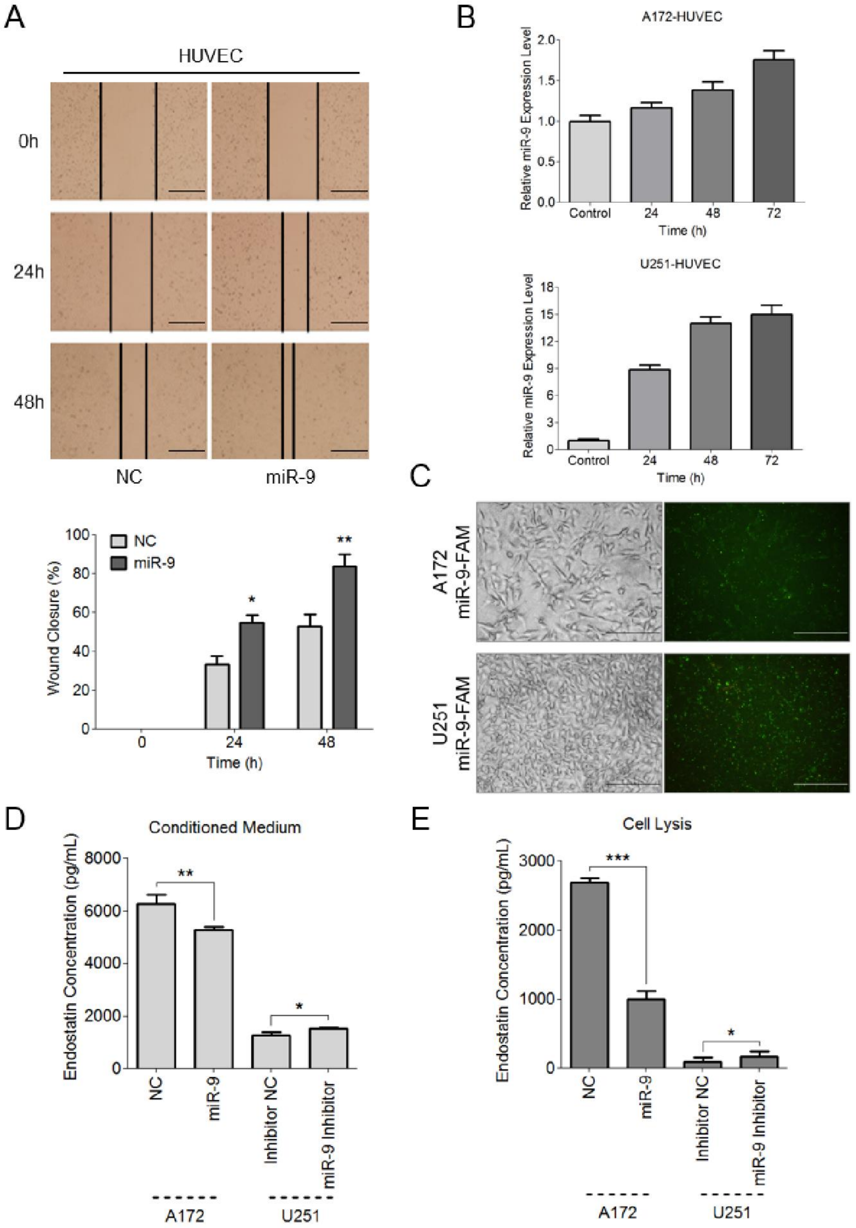

Figure S3. MiR-9 is involved in the regulation of basic biological behaviors of the HUVECs. (A) Transfection efficiency of the miR-9 mimic/NC was measured by qRT-PCR analysis in HUVECs. Data are represented as the mean ± s.d. (***P < 0.001; n = 3 independent experiments). (B and C) Flow cytometry analysis was used to detect the apoptosis (B) and cell cycle distribution (C) of HUVEC miR-9 mimic/NC cells. Data are presented as the mean ± s.d. (N.S, no significance; n = 3 independent experiments). (D) Wound-healing assay (upper) was applied to detect the migration ability of the HUVEC miR-9 mimic/NC cells at 0, 24 and 48 h. Histogram is used for statistical analysis (lower). Scale bars represent 200 μm. Data are shown as the mean ± s.d. (*P < 0.05 and **P < 0.01; n = 3 independent experiments).

Figure S4.

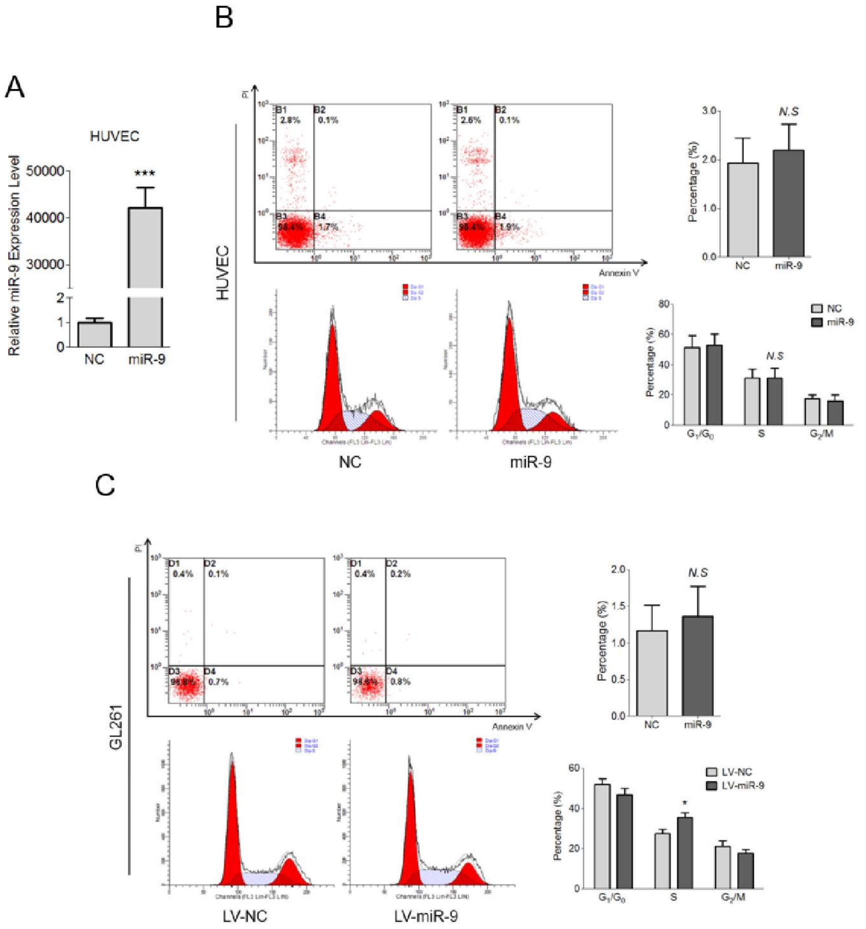

Figure S4. MiR-9 acts as an angiogenesis inducer that is secreted from glioma cells and taken in by the HUVECs.

(A) Expression levels of the miR-9 in the HUVECs that were cultured with the conditional medium harvested at 24, 48 and 72 h from A172 (left) and U251 (right) cells were detected by qRT-PCR, respectively. Data are presented as the mean ± s.d.. (B) The FAM-labeled miR-9 transfected A172 and U251 cells were observed under a white and fluorescence microscope after 24 h. Scale bars represent 100 μm. (C and D) Expressions of endostatin in the conditional medium (C) along with cell lysates (D) of A172 miR-9 mimic/NC and U251 miR-9 inhibitor/NC cells were analyzed by ELISA. Error bars represent the s.d. (*P < 0.05, **P < 0.01 and ***P < 0.001; n = 3 independent experiments).

Figure S5.

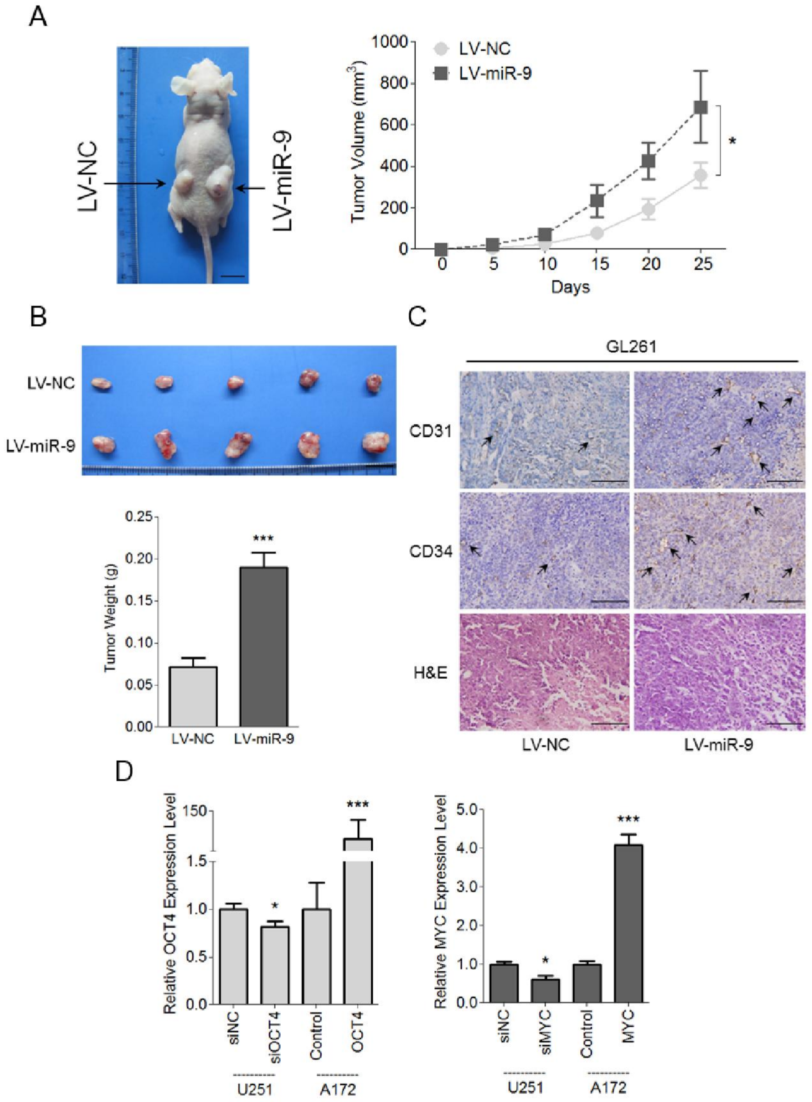

Figure S5. MiR-9 promotes the glioma growth and novel vessel formation in vivo.

(A) Flow cytometry analysis were used to detect the cell apoptosis (upper) and cell cycle (lower) of the lentivirus infected GL261 LV-miR-9 and GL261 LV-NC cells. Data are represented as the mean ± s.d. (N.S, no significance; *P < 0.05; n = 3 independent experiments). (B) Model of the subcutaneous injection of GL261 LV-miR-9/NC cell-injected athymic mice. Scale bars represent 1 cm. (C) Separated neoplasms (left) and tumor growth curve (right) of GL261 LV-miR-9/NC cell-injected athymic mice were exhibited. Tumor volume data were recorded once every five days. Scale bars represent 1 cm. Data are represented as the mean ± s.d. (*P < 0.05; n = 5 independent experiments). (D) Weights of the neoplasms were examined, respectively. Data are shown as the mean ± s.d. (***P < 0.001; n = 5 independent experiments). (E) The representative CD31 and CD34 staining was determined by IHC analysis of the GL261 LV-miR-9/NC cell-injected tumor tissue sections. Positive signals are marked by black arrows. Scale bars represent 200 μm. (F) The transfection efficiency of the MYC/OCT4 overexpression in A172 cells and knockdown in U251 cells was assessed by qRT-PCR analysis. Data are presented as the mean ± s.d. (*P < 0.05 and ***P < 0.001; n = 3 independent experiments).

Figure S6.

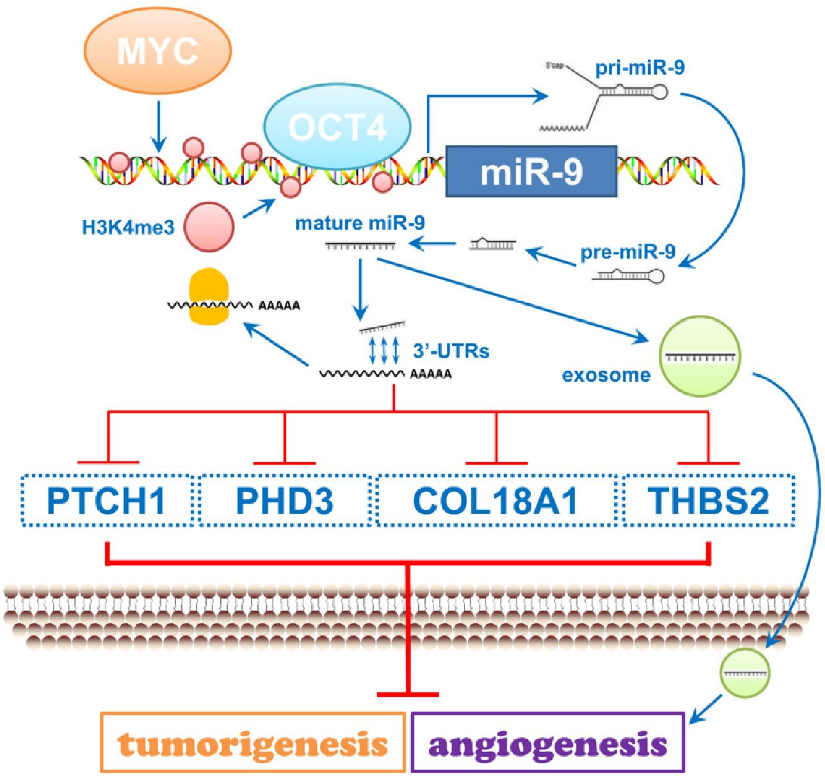

Figure S6. Pattern diagram that summarize the regulatory model in our study.

In human glioma cells, two stem cell factors, MYC and OCT4, are capable of directly binding to the promoter region of miR-9 to trigger its transcription. The mature miR-9 can directly suppress COL18A1, THBS2, PTCH1 and PHD3 by interacting with their mRNA 3'-UTRs. MiR-9 can also be secreted by the glioma cells via exosomes and is subsequently absorbed by the vascular endothelial cells to help achieve neovascularization. Thus, miR-9 triggers the tumorigenesis and angiogenesis during glioma development and progression.
